# Proteomic and histopathological characterisation of sicca subjects and primary Sjögren’s syndrome patients reveals promising tear, saliva and extracellular vesicle disease biomarkers

**DOI:** 10.1186/s13075-019-1961-4

**Published:** 2019-07-31

**Authors:** Lara A. Aqrawi, Hilde Kanli Galtung, Eduarda M. Guerreiro, Reidun Øvstebø, Bernd Thiede, Tor Paaske Utheim, Xiangjun Chen, Øygunn Aass Utheim, Øyvind Palm, Kathrine Skarstein, Janicke Liaaen Jensen

**Affiliations:** 1 Department of Oral Surgery and Oral Medicine, University of Oslo, Oslo, Norway; 2 Department of Oral Biology, Faculty of Dentistry, University of Oslo, Oslo, Norway; 3 Department of Medical Biochemistry, Oslo University Hospital, Oslo, Norway; 4 Department of Biosciences, University of Oslo, Oslo, Norway; 5 Department of Plastic and Reconstructive Surgery, Oslo University Hospital, Oslo, Norway; 6 The Norwegian Dry Eye Clinic, Oslo, Norway; 7 Department of Rheumatology, Oslo University Hospital, Oslo, Norway; 8 Gade Laboratory for Pathology, Department of Clinical Medicine, University of Bergen, Bergen, Norway; 9 Department of Pathology, Haukeland University Hospital, Bergen, Norway

**Keywords:** Sjögren’s syndrome, Autoimmunity, Sicca subjects, Proteomics, Biomarkers, Innate immunity, Adaptive immunity, Tears, Saliva, Extracellular vesicles

## Abstract

**Background:**

Mononuclear cell infiltration of exocrine glands, production of Ro/SSA and La/SSB autoantibodies, along with oral and ocular dryness, are characteristic features of primary Sjögren’s syndrome (pSS). Non-SS sicca subjects, an underexplored group in relation to pSS, display similar sicca symptoms, with possible mild signs of inflammation in their salivary glands, yet with no serological detection of autoantibody production. In this study, we investigated inflammatory manifestations in the salivary gland tissue, tear fluid and saliva of non-SS subjects, as compared to pSS patients and healthy individuals.

**Methods:**

Fifteen non-SS, 10 pSS and 10 healthy subjects were included in the analyses. Histological evaluation of salivary gland biopsies was performed. Liquid chromatography-mass spectrometry (LC-MS) was conducted on tear fluid and stimulated whole saliva, and proteomic biomarker profiles were generated. Extracellular vesicle (EVs) isolation and characterisation from both fluids were also combined with LC-MS. The LC-MS data were analysed for quantitative differences between patient and control groups using Scaffold. Database for Annotation, Visualization and Integrated Discovery (DAVID) and Functional Enrichment Analysis Tool (FunRich) were applied for functional analyses.

**Results:**

Histopathological evaluation of salivary gland biopsies showed implications of milder inflammation in non-SS subjects through mononuclear cell infiltration, fibrosis and fatty replacement, as compared to pSS patients. Although unaffected in the non-SS group, upregulation of proinflammatory pathways and proteins involved in ubiquitination (LMO7 and HUWE1) and B cell differentiation (TPD52) were detected in tear fluid of pSS patients. Moreover, overexpression of proteins STOM, ANXA4 and ANXA1, regulating cellular innate and adaptive immunological pathways, were further identified in EVs from tear fluid of pSS patients. Finally, whole saliva and EVs isolated from whole saliva of pSS patients expressed proteins vital for innate MHC class I cellular regulation (NGAL) and T cell activation (CD44).

**Conclusions:**

Non-SS sicca subjects may show implications of mild inflammation in their glandular tissue, while their protein profile was strikingly more similar to healthy controls than to pSS patients. Hence, the tear and salivary biomarkers identified could be implemented as potential non-invasive diagnostic tools that may aid in increasing diagnostic accuracy when evaluating non-SS subjects and pSS patients and monitoring disease progression.

**Electronic supplementary material:**

The online version of this article (10.1186/s13075-019-1961-4) contains supplementary material, which is available to authorized users.

## Background

Primary Sjögren’s syndrome (pSS) is a systemic rheumatic autoimmune disease that is characterised by chronic inflammation, autoantibody production and destruction of exocrine glands through mononuclear cell infiltration. The primary target organs are the lacrimal and salivary glands [1, 2], resulting in reduced secretion of tears and saliva [3]. The main classification criteria used today for pSS are the American-European Consensus Group (AECG) criteria from 2002 [4, 5] and the American College of Rheumatology (ACR) criteria from 2012 [6]. In addition to evaluating symptoms of ocular and oral dryness, assessing the secretory ability of exocrine glands, and screening for anti-Ro and anti-La autoantibodies, minor salivary gland biopsies are evaluated for mononuclear cell infiltration, also known as focus scoring [7]. This routine histological assessment has been employed to describe salivary gland involvement in SS [8, 9], where a biopsy of focus score ≥ 1 (i.e., ≥ 1 foci per 4 mm^2^) is considered positive. In some cases, subjects may display sicca symptoms and may show some mild infiltration of mononuclear cells in their exocrine glands, yet serologically no autoantibody production is detected [10]. Hence, these underexplored non-SS sicca subjects represent an interesting study group when compared to both pSS patients and healthy individuals, since they lack the characteristic features for attaining the pSS diagnosis, yet possess the symptomatic characteristics of ocular and oral dryness nonetheless. Whether these alterations are the result of a different disease course stands to be determined.

Interestingly, the destruction of salivary gland tissue through the deregulated infiltration and proliferation of lymphocytes may also lead to the formation of ectopic germinal centre (GC)-like structures in approximately 20% of pSS patients [11–14]. It is also commonly accompanied by the development of both adipose (fatty) tissue and fibrosis [15]. The presence of adipose tissue replacement has also been observed in non-SS sicca subjects, yet to a lesser degree [16, 17]. Hence, evaluating the degree of adipose tissue replacement as part of routine salivary gland assessment has been suggested as an additional helpful tool when classifying pSS patients [16].

In view of currently available diagnostic tools for pSS, there is an unmet need for the incorporation of non-invasive, more accurate diagnostics. Studying the proteome of biological fluids and screening for disease-specific biomarkers [18] through liquid chromatography-mass spectrometry (LC-MS) [19, 20] has therefore been in focus over the last decades. Both saliva [18, 21–27] and tear fluid [28, 29] have previously been analysed to identify potential biomarkers for SS. Moreover, salivary and tear fluid samples can easily be obtained using a simple, non-invasive, and fairly safe procedure that also permits repetition and multiple collections. The majority of proteomic studies of SS have chosen saliva as the ideal biological fluid for performing LC-MS analyses, under both stimulated and unstimulated conditions. As a result, several common biomarkers for SS have been identified, including highly abundant immune-system-related molecules, secretory proteins, enzymes, and cytokines [19, 26, 30]. Examples of such biomarkers include β-2 microglobulin (B2MG), Neutrophil gelatinase-associated lipocalin (LCN2), Lymphocyte-specific protein 1 (LSP1), interleukin-4 (IL-4), IL-5, and Clusterin (CLU), displaying molecules active in both innate and adaptive immunity.

Various separation techniques can also be coupled with proteomic analyses, in order to isolate cellular components of interest when screening for disease biomarkers. Extracellular vesicles (EVs) are an example of such cellular components, comprising of exosomes (size < 100 nm), microvesicles (size 100–1000 nm), and apoptotic bodies (size 1000–5000 nm) [31]. Interestingly, EVs can be separated and purified through different approaches, including size-exclusion chromatography [32–34]. They are regarded as important mediators of intercellular communication that can influence recipient cell functions [35–37]. For instance, EVs can act as inducers of pro-inflammatory signals on the innate immune system during infections [38]. Patients with autoimmune diseases have also displayed increased levels of EVs associated with inflammation [39] and complement activation [40]. Consequently, various cell types of the innate immune system are known to release EVs, including natural killer (NK) cells [41], macrophages [42], monocytes and dendritic cells [43].

We have previously applied LC-MS using samples of stimulated whole saliva and tear fluid from patients with pSS and healthy controls, in combination with EV isolation, which resulted in the detection of potential novel disease biomarkers [29, 44]. To date, non-SS sicca subjects remain understudied within the field of proteomics. Still, they represent an interesting analytical group, in relation to pSS, that displays the common symptoms of dry eyes and dry mouth, and may also show mild signs of inflammation in their salivary gland tissue, yet remain serologically autoantibody-negative. Whether these discrepancies are the result of a different disease trajectory remains to be explored. Hence, we wished to further investigate patterns of chronic inflammation in the salivary gland tissue, tear fluid and saliva of these non-SS sicca subjects. By applying histopathological assessment of minor salivary gland biopsies, in combination with LC-MS on tear fluid and saliva, coupled with EV isolation, we aimed to gain insight into the cellular processes propagating disease and delineate whether this underexplored group of non-SS subjects behaves more like pSS patients or healthy controls on a glandular and protein level. Accordingly, additional biomarkers may also be identified, and in turn implemented as potential non-invasive diagnostic tools that can aid in increasing diagnostic accuracy when evaluating non-SS sicca subjects and patients with pSS, in accordance with the AECG and ACR criteria.

## Methods

### Study population

Fifteen non-SS sicca subjects, 10 pSS patients that fulfilled the AECG classification criteria from 2002 [4] and 10 age- and gender-matched healthy controls participated in the current study. The subjects in the non-SS group possessed dry eye and dry mouth symptoms, yet did not fulfil the classification criteria for pSS due to negative anti-SSA/SSB serology and a focus score < 1 in their evaluated salivary gland biopsies. These biopsies were collected at the Department of Oral Surgery and Oral Medicine, University of Oslo (JLJ), and evaluated at the Gade Laboratory for Pathology, University of Bergen (KS). Following recruitment at the Department of Rheumatology, Oslo University Hospital, the pSS patients, along with the non-SS sicca subjects and volunteering healthy controls, were all referred to the Norwegian Dry Eye Clinic, Oslo, and the Dry Mouth Clinic, Oslo. At these clinics, participants underwent a thorough ocular and oral examination, followed by tear fluid and stimulated saliva sample collection, as described below. A detailed explanation of the study aim and protocols was provided to the recruited subjects upon enrolment. Written informed consent was also obtained from the participants, and the Regional Medical Ethical Committee of South-East Norway approved the study (REK 2015/363). Medical records and clinical data of the pSS patients were attained from the Department of Rheumatology, Oslo University Hospital. The demographics of the non-SS and pSS subjects participating in the current study are presented in Tables 1 and 2.

**Table 1:** Clinical characteristics of non-SS subjects included in the proteomics analysis

Patient no.	Age	Gender	Anti-SSA*	Anti-SSB*	Focus score**	FI score***	Schirmer test****	Saliva secretion *****	Dry mouth	Dry eyes
1	71	F	–	–	< 1	1	+	+	+	+
2	33	F	–	–	0	1	+	NT	+	+
3	48	F	–	–	0	0	+	+	+	+
4	65	F	–	–	0	2	+	+	+	+
5	39	F	–	–	0	0	+	+	+	+
6	44	F	–	–	0	0	–	+	+	+
7	30	F	–	–	0	0	–	+	+	+
8	56	F	–	–	0	1	+	+	+	+
9	41	F	–	–	0	0	+	+	+	+
10	50	F	–	–	0	0	+	+	+	+
11	47	F	–	–	0	1	+	–	+	+
12	64	F	–	–	< 1	0	+	+	+	+
13	73	F	–	–	0	2	+	+	+	+
14	59	F	–	–	< 1	0	+	+	+	+
15	51	F	–	–	< 1	–	+	+	+	+

*F* female, *FI* fatty infiltration, *NT* not tested

*Autoantibody production was assessed by ELISA

**Values are the number of focal infiltrates/4mm^2^ tissue area containing > 50 mononuclear cells

***The degree of fatty infiltration was assessed and the sections were scored blindly, where no or little fatty infiltration = 0, moderate = 1, and prominent = 2

****Values are in mm/5 min; normal flow > 5 mm/5 min. ‘+’ indicates dryness and tear secretion ≤ 5 mm/5 min

*****Values are in ml/15 min; normal flow > 1.5 ml/15 min. ‘+’ indicates dryness and unstimulated whole saliva secretion ≤ 1.5 ml/15 min

**Table 2:** Clinical characteristics of pSS patients included in the study

Patient no.	Age	Gender	Anti-SSA*	Anti-SSB*	Focus score**	GC	FI score***	Schirmer test****	Saliva secretion *****	Dry mouth	Dry eyes
1	48	F	+	+	–	–	–	+	+	+	+
2	59	F	+	+	–	–	–	+	+	+	+
3	52	F	+	+	8	+	1	+	+	+	+
4	54	F	+	–	1	–	2	+	+	–	+
5	60	F	+	+	3	+	1	+	+	–	–
6	64	F	+	–	0	–	1	+	+	+	+
7	55	F	+	–	0	–	2	+	+	+	+
8	50	F	+	+	–	–	–	+	–	+	+
9	35	F	+	+	3	+	0	+	+	–	–
10	75	F	+	+	–	–	–	+	–	+	+

*F* female, *GC* germinal centres, *FI* fatty infiltration

*Autoantibody production was assessed by ELISA

**Values are the number of focal infiltrates/4mm^2^ tissue area containing > 50 mononuclear cells

***The degree of fatty infiltration was assessed and the sections were scored blindly, where no or little fatty infiltration = 0, moderate = 1, and prominent = 2

****Values are in mm/5 min; normal flow > 5 mm/5 min. ‘+’ indicates dryness and tear secretion ≤ 5 mm/5 min

*****Values are in ml/15 min; normal flow > 1.5 ml/15 min. ‘+’ indicates dryness and unstimulated whole saliva secretion ≤ 1.5 ml/15 min

### Histopathological evaluation of minor salivary gland biopsies

Routine haematoxylin and eosin-stained sections from minor salivary gland biopsies of the non-SS and pSS subjects included in the study were evaluated using a light microscope (Leica, DMLB, Leica Microsystems Wetzlar, Germany). Both mononuclear cells in focal infiltrates and those located interstitially, i.e., in close proximity to the acinar or ductal epithelium, were analysed. Additionally, other forms of tissue damage, including fibrosis, in the same area were also investigated. Furthermore, these salivary gland sections were scored blindly for the presence of fatty infiltration, as previously described [16, 17]. Depending on the degree of fat deposition either numbers 0, 1 or 2 was assigned for each category during the assessment, where 0 was regarded negative to little, while 1 was considered moderate, and 2 signified prominent fatty infiltration*.*

### Tear fluid and saliva collection

Participants underwent a thorough ocular surface examination at the Norwegian Dry Eye Clinic, and a detailed oral examination at the Dry Mouth Clinic, where tear fluid and stimulated whole saliva were collected, respectively, as previously described [29, 45]. In brief, the tear fluid was collected from both eyes using a Schirmer tear test strip (HAAG-STREIT, Essex, UK) to produce a minimum combined total of 10 mm of tear volume, that was then transferred to 500 μl of 0.1 μm filtered phosphate-buffered saline (PBS) (Gibco, pH 7.4, ThermoFisher Scientific, Oslo, Norway). Additionally, stimulated whole saliva was collected on ice from all participants, while chewing on a paraffin block (Paraffin Pellets, Ivoclor Vivadent, Shaen, Lichtenstein) for 5 min. Only patients producing ≥ 800 μl of stimulated whole saliva were included in the study. All tear fluid and saliva samples were then stored at − 80 °C.

### Extraction of EVs from tear fluid and saliva

EVs were isolated from tear fluid and stimulated whole saliva using size-exclusion chromatography, as described previously [33]. In brief, due to the low volume of tear fluid collected from the individual pSS patients, tear fluid of Schirmer strips from all non-SS subjects, pSS patients and healthy controls were pooled into three groups, respectively, concentrated to 200 μl using Amicon Ultra-4 columns, and then adjusted to a volume of 1 ml with 0.1 μm filtered PBS. Saliva samples from all participants were centrifuged at 300 rpm for 10 min to remove debris, and then diluted 1:2 with 0.1 μm filtered PBS. A qEV size-exclusion chromatography column (iZON Science, Oxford, UK) was equilibrated by washing the column with 15 ml of 0.1 μm filtered PBS. Samples were then added to the equilibrated qEV size-exclusion chromatography column, and 16 fractions, each 500 μl in volume, were collected by continuously adding 0.1 μm filtered PBS to the column. A new column was used for each sample. The eluted fractions 8–10 (containing the majority of microvesicles and exosomes present in the samples) were concentrated for 80 min at 30 °C in an Eppendorph concentrator 5301 (Eppendorph AG, Hamburg, Germany) and collected into a joint fraction. The protein concentration was then determined using Qubit Fluorometric Quantitation (ThermoFisher Scientific, Oslo, Norway). A volume of the tear fluid, the diluted stimulated whole saliva (100 μl), the joint fractions from the pooled tear samples, and from each saliva sample were then sent for proteomic analysis while preserved on dry ice.

### Characterisation of EVs

In order to characterise the isolated EVs, nanoparticle tracking analysis and immunoaffinity capture for detection of CD9 positive EVs were conducted on joint fractions from saliva and tears as previously described [29]. In brief, the nanoparticle tracking analysis determined the size distribution and concentration of the respective EVs using a NanoSight NS500 instrument (Malvern Instruments Ltd., Malvern, UK) [46]. The hydrodynamic diameter of the particles in each sample was calculated by the NTA 3.0 software (Malvern Instruments, Malvern, UK). Additionally, immunoaffinity capture for detection of CD9 positive EVs was performed using the Exosome Human CD9 Flow Detection Kit (Dynal®, ThermoFisher Scientific, Oslo, Norway) and flow cytometry with BD Accuri™ C6 Cytometer (BD Biosciences, Oslo, Norway). Median fluorescence intensity (MFI) was reported as a signal to noise (S/N) ratio to isotype control from a total of 300 singlet events. A summary of the measurements obtained from the nanoparticle tracking analysis and the flow cytometry analyses in tear fluid and saliva is presented in Table 3.

**Table 3:** Characterisation of EVs in saliva and tears

	Mean particle size* (nm)	Particles/mL*	CD9 + EVs** S/N ratio MFI
Tear fluid			
Pool of patients with pSS (*n* = 9)	255 ± 40	5.0 E+08	1.04
Pool of patients with non-SS (*n* = 14)	204 ± 8	1.2 E+09	1.22
Pool of controls (*n* = 10)	215 ± 9	7.1 E+08	1.20
Saliva			
Patients with pSS (*n* = 9)	233 ± 17	1.9 E+10 ± 0.7E+9	4.32 ± 1.19
Patients with non-SS (*n* = 14)	231 ± 13	1.0 E+10 ± 1.6E+9	3.98 ± 0.57
Controls (*n* = 10)	264 ± 6	7.9 E+9 ± 1.6E+9	3.22 ± 0.98

*Nanoparticle tracking analysis was conducted on EV joint fractions from pooled tear fluid (*n* = 9 pSS, *n* = 14 non-SS and *n* = 10 controls) and whole saliva (*n* = 9 pSS, *n* = 14 non-SS and *n* = 10 controls), to determine mean particle size of microvesicles and exosomes (nm ± SEM), in addition to concentrations of EVs (particles/ml ± SEM)

**Detection of CD9+ EVs from joint fractions of pooled tear fluid (*n* = 9 pSS, *n* = 14 non-SS and *n* = 10 controls), and whole saliva (*n* = 9 pSS, *n* = 14 non-SS and *n* = 10 controls) was performed by immunoaffinity capture using anti-CD9 coated magnetic beads followed by flow cytometry analysis. The results were reported as signal to noise (S/N) ratios of median fluorescence intensity (MFI)

### Protein profiling by LC-MS

Proteomics analysis was performed on saliva and tears from non-SS sicca subjects, pSS patients, and healthy controls before and after isolation of EVs, as previously described [29]. In brief, samples were diluted with ice-cold acetone, vortexed, precipitated overnight at − 20 °C, centrifuged at 16000*g* for 20 min at 4 °C (Centrifuge 5415R, Eppendorf, Hamburg, Germany), and then re-dissolved in 50 μl of a mixture of 6 M urea and 100 mM ammonium bicarbonate (pH 7.8), followed by reduction and alkylation of cysteines. The alkylation reaction was quenched, and the proteins were digested with 10 μg of trypsin for 16 h at 37 °C to generated peptides that were then purified using an OMIX C18-micro SPE (Agilent, Santa Clara, CA, USA) and dried using a Speed Vac concentrator (Concentrator Plus, Eppendorf, Hamburg, Germany). These tryptic peptides were analysed using an Ultimate 3000 RSLCnano-UHPLC system connected to a Q Exactive mass spectrometer (Thermo Fisher Scientific, Bremen, Germany) and a nano electrospray ion source.

For liquid chromatography separation, an Acclaim PepMap 100 column (C18, 2 μm beads, 100 Å, 75 μm inner diameter, 50 cm length) (Dionex, Sunnyvale CA, USA) was used. A flow rate of 300 nL/min was employed with a solvent gradient of 4–35% B in 60 min. Solvent A was 0.1% formic acid, and solvent B was 0.1% formic acid/90% acetonitrile.

The mass spectrometer was operated in the data-dependent mode to automatically switch between MS and MS/MS acquisition. Survey full-scan MS spectra (from m/z 400 to 2000) were acquired with the resolution *R* = 70,000 at m/z 200, after accumulation to a target of 1e6. The maximum allowed ion accumulation times were 60 ms. The method used allowed sequential isolation of up to the ten most intense ions, depending on signal intensity (intensity threshold 1.7e4), for fragmentation using higher-energy collisional induced dissociation (HCD) at a target value of 1e5 charges, NCE 28, and a resolution *R* = 17,500. Target ions already selected for MS/MS were dynamically excluded for 30 s. The isolation window was m/z = 2 without offset. For accurate mass measurements, the lock mass option was enabled in MS mode.

Finally, data were acquired using Xcalibur v2.5.5, and raw files were processed to generate peak lists in Mascot generic format (*.mgf) using ProteoWizard release version 3.0.331. Database searches were performed using Mascot in-house version 2.4.0 to search the SwissProt database (Human, 20,279 proteins), as before [29].

### LC-MS data processing and statistical analysis

In order to validate MS/MS-based peptide and protein identifications, Scaffold (version Scaffold_4.4, Proteome Software Inc., Portland, OR) was used, as before [29]. Here, peptide identifications were accepted if they could be established at greater than 95.0% probability by the Scaffold Local FDR algorithm, while protein identifications were accepted if they could be established at greater than 99.0% probability. For label-free quantification, the entire MS2 total ion current (TIC) across all biological replicates was evaluated using t-test (*p* < 0.05). For functional analysis of the proteomics data, Database for Annotation, Visualization and Integrated Discovery (DAVID) (v 6.7, https://david.ncifcrf.gov) and Functional Enrichment Analysis Tool (FunRich) (http://www.funrich.org/) were applied. Tear fluid, stimulated whole saliva, and EVs (joint fractions) were analysed individually, comparing pSS patients with the non-SS sicca subjects and the healthy controls, correspondingly. DAVID was applied, using a False Discovery Rate (FDR) with a maximum 5% cut-off, in order to delineate specific cellular pathways involving these upregulated proteins in the pSS patients, while FunRich was used to visualise the percentage of proteins involved in each of these upregulated signalling pathways.

## Results

### Histopathological evaluation of minor salivary gland biopsies shows patterns of chronic inflammation in the target organ of non-SS sicca subjects

**Fig. 1:**
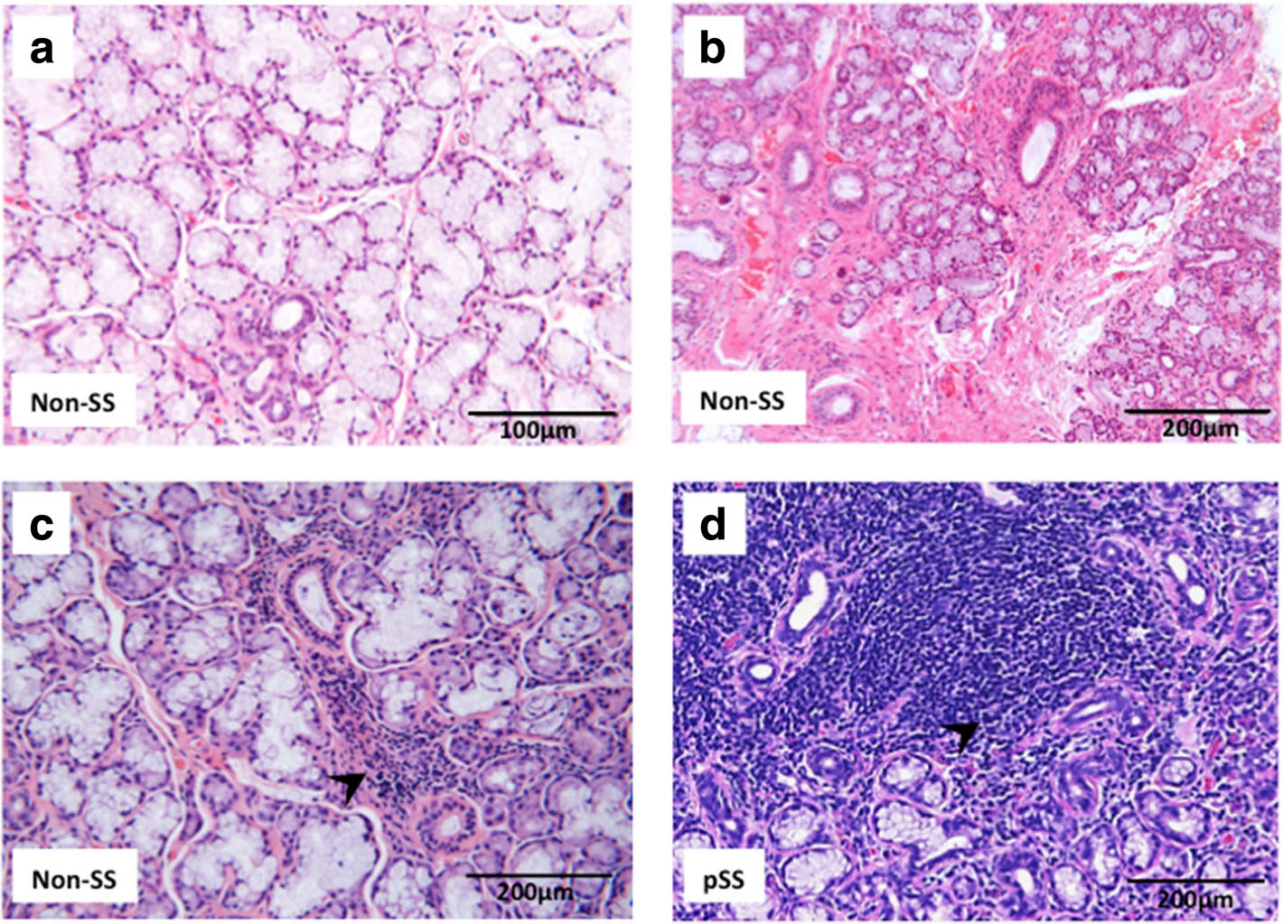
Histopathological evaluation of minor salivary gland biopsies shows implications of inflammation in the target organ of non-SS sicca subjects. Haematoxylin and eosin staining of minor salivary gland biopsies taken from the non-SS and pSS subjects included in the study allowed the evaluation for mononuclear cell infiltration, fibrosis, and the presence of fatty infiltration in their salivary gland tissue. **a** Non-SS subject with normal salivary gland morphology. **b** Non-SS individual with fibrosis in the salivary gland tissue. **c** Non-SS participant with mild focal inflammation in the salivary gland and a focus score < 1. **d** Salivary gland biopsy of a pSS patient with a focus score value of 3 and GC-like structure within the focal infiltrate. Areas of inflammation are indicated by black arrow

In order to account for the morphological patterns of chronic inflammation in minor salivary gland biopsies of the non-SS and pSS subjects included in the study, the sections were evaluated for mononuclear cell infiltration, other form of tissue damage including fibrosis, and for the presence of fatty infiltration [16, 17]. Interestingly, 27% of the non-SS subjects showed some focal chronic inflammation in their salivary gland tissue and had a focus score value of < 1. Also, 67% of those pSS patients that have had their biopsies taken had a positive focus score, ranging from 1 to 8, and 50% of these biopsies were also GC positive. Additionally, 83% of pSS patients had a positive fatty infiltration score in their salivary glands; where 17% had a fatty infiltration score of 0, 50% had a score of 1, and 33% had a score of 2. Meanwhile, 40% of the non-SS sicca participants also showed fatty infiltration in their biopsies (Tables 1 and 2). An illustration of such focal chronic inflammation and fibrosis detected in the non-SS subjects, as compared to pSS patients, is presented in Fig. 1.

### Upregulation of proinflammatory pathways and proteins involved in ubiquitination and B cell differentiation detected in tear fluid of pSS patients, as compared to both non-SS sicca subjects and healthy controls

**Fig. 2:**
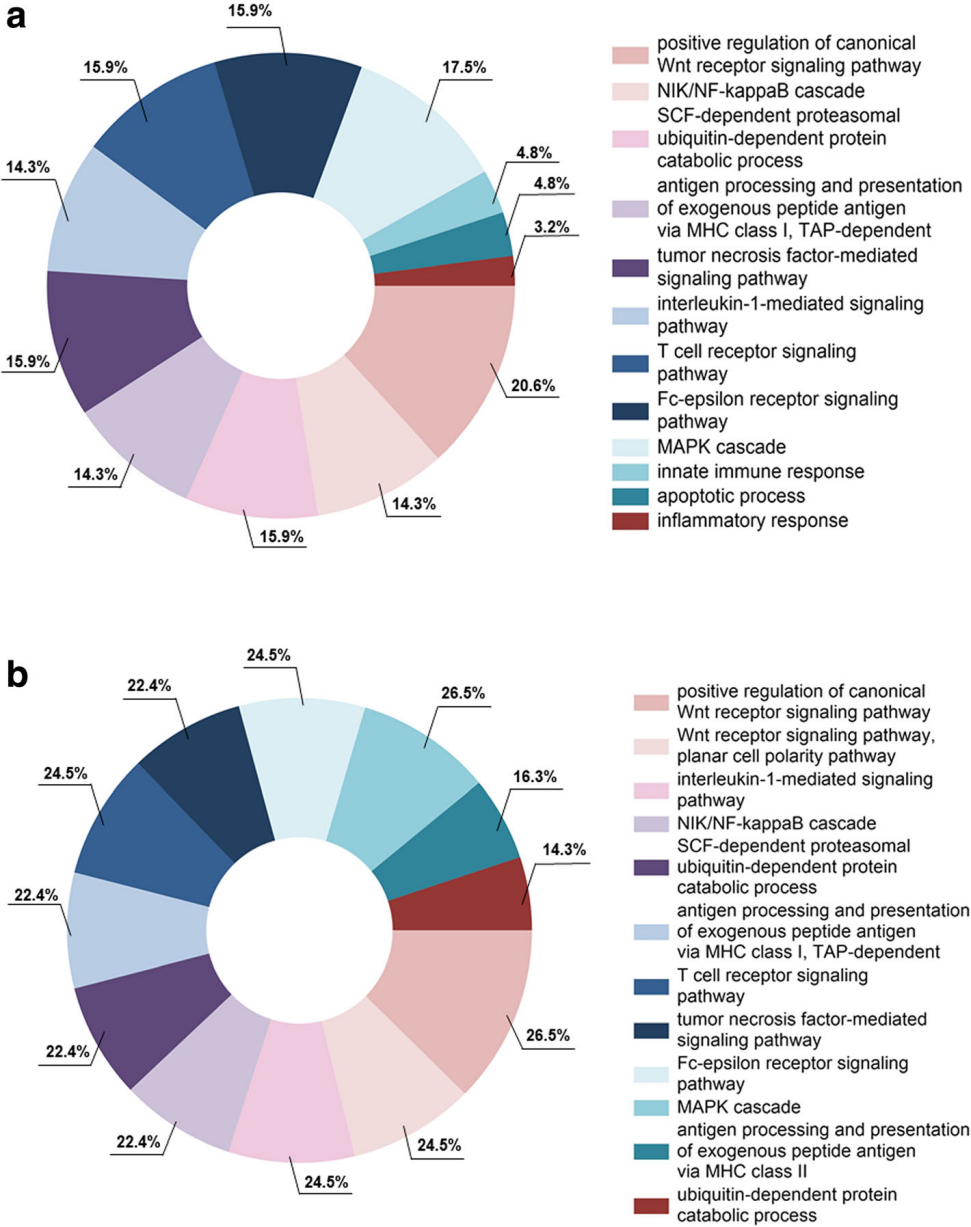
Upregulation of proinflammatory pathways detected in tear fluid of pSS patients. For functional analysis of the proteomics data, DAVID (v 6.7, https://david.ncifcrf.gov) was applied using a FDR with a maximum 5% cut-off, and cellular processes for the upregulated proteins in the pSS patients were identified. FunRich (http://www.funrich.org/) was then used to visualise the fraction of proteins involved in each of these upregulated signalling pathways. **a** Upregulated signalling pathways identified in the pSS patients, as compared to non-SS sicca controls. **b** Comparing pSS patients to healthy controls helped detect similar cellular processes as with the non-SS subjects, affecting both innate and adaptive immunological processes. Percentage values indicate the amount of overexpressed proteins involved in upregulating each of the cellular processes identified

Performing LC-MS on tear fluid from 15 non-SS sicca subjects, 10 pSS patients, and 10 healthy controls helped identify significantly upregulated proteins with *p* values < 0.05 that were distinguished using spectral counts (Additional file 4: Table S1 and Additional file 5: Table S2). These upregulated proteins were further analysed using DAVID, and cellular processes for the upregulated proteins in the pSS patients were detected. Upregulated signalling pathways identified in the pSS patients, as compared to non-SS sicca controls, included Wnt receptor signalling (20.6%), MAP kinase cascade, ubiquitination, tumour necrosis factor (TNF)-mediated signalling, T cell receptor signalling, Fc receptor signalling, NF-kappa B cascade, MHC class I antigen processing and presentation, IL-1 mediated signalling, in addition to general innate immune responses, apoptotic processes, and inflammatory responses, in descending order, as indicated by the percent values of the upregulated proteins involved in each cellular process (Fig. 2a). Similarly, when comparing these pSS patients to the healthy controls, the same cellular processes were observed as a result of upregulated proteins in the patients, with the addition of MHC class II antigen processing and presentation, and catabolic processes (Fig. 2b).

Since proteins found upregulated in the pSS patient group could be the most promising candidates for potential disease biomarkers, we also considered the number of spectral counts in our analyses. Accordingly, the three most upregulated proteins in pSS patients that are involved in immunological reactions, when compared to both non-SS subjects and healthy controls, were LIM domain only protein 7 (LMO7), E3 ubiquitin-protein ligase HUWE1 (HUWE1) and Tumour protein D52 (TPD52), in descending order. The most upregulated protein in tear fluid of patients with pSS, namely LMO7, is involved in ubiquitination and cell adhesion. Moreover, HUWE1, also mediates ubiquitination, in addition to neutrophil degranulation and cell differentiation. Lastly, TPD52 plays a central role in regulating B cell differentiation (Table 4, Additional file 1: Figure S1).

**Table 4:** Highly upregulated proteins in tear fluid of pSS patients

Number	Gene	Related protein*	Classification and function**
Non-SS vs. pSS			
1	LMO7	LIM domain only protein 7	Ubiquitination, cell signalling, cell adhesion
2	HUWE1	E3 ubiquitin-protein ligase HUWE1	Mediates ubiquitination, neutrophil degranulation, cell differentiation
3	TPD52	Tumour protein D52	B cell differentiation, cell proliferation, Ca^2+^-signalling
Controls vs. pSS			
1	LMO7	LIM domain only protein 7	Ubiquitination, cell signalling, cell adhesion
2	HUWE1	E3 ubiquitin-protein ligase HUWE1	Mediates ubiquitination, neutrophil degranulation, cell differentiation
3	TPD52	Tumour protein D52	B cell differentiation, cell proliferation, Ca^2+^-signalling

*The three most upregulated immunological proteins in whole saliva of pSS patients deviating in replicates, i.e. number of individuals (frequency), and spectral counts, as identified by proteomics analysis and Scaffold (v 4.4.6, http://www.proteomesoftware.com/products/scaffold/)

**The classification and functions of the proteins presented were identified using publicly available databases, such as UniProt (http://www.uniprot.org)

### Overexpression of proteins regulating cellular innate and adaptive immunological pathways detected in EVs from tear fluid of pSS patients, utilising non-SS sicca subjects and healthy individuals as controls

**Fig. 3:**
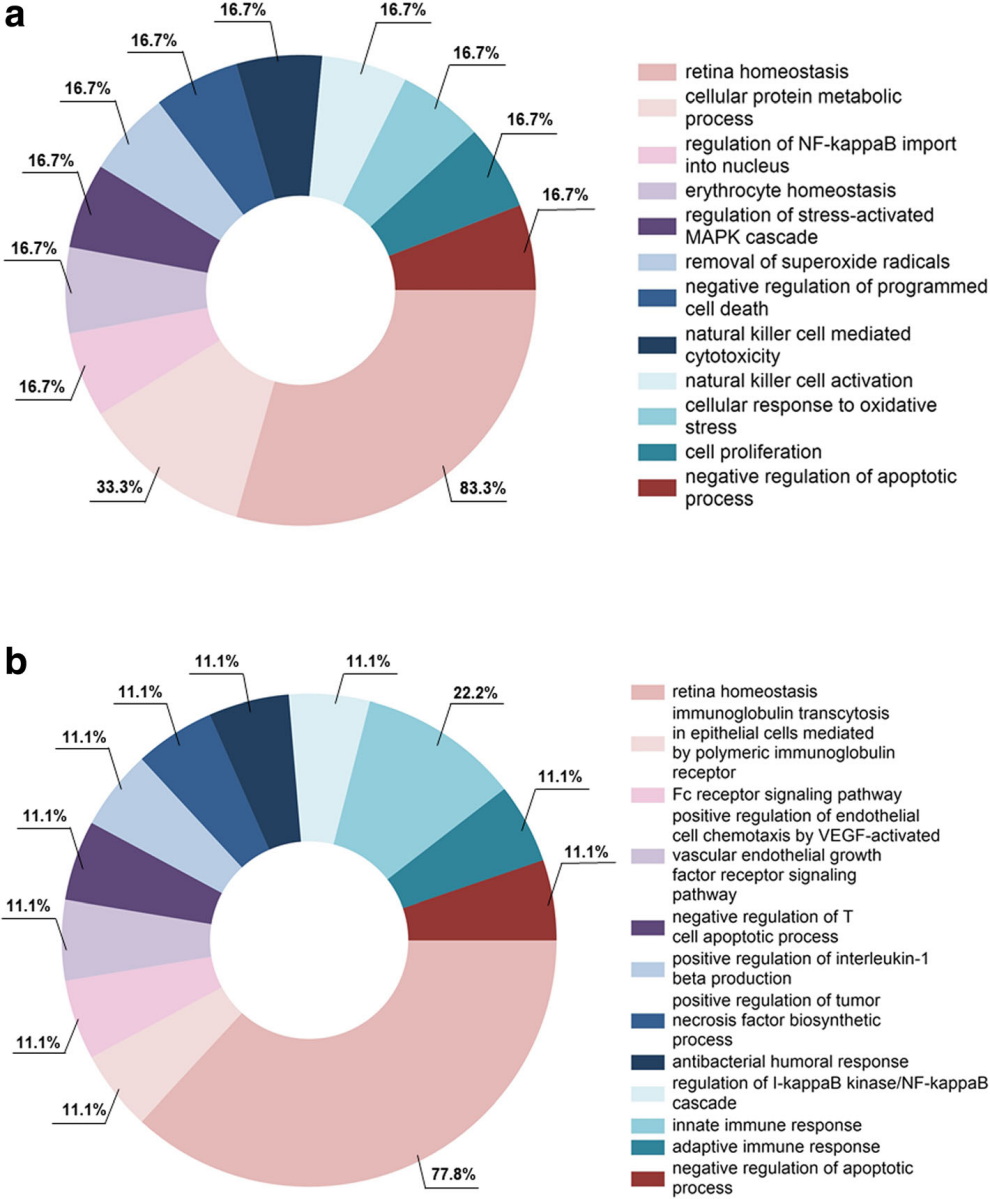
Overexpression of proteins regulating cellular innate and adaptive immunological pathways detected in EVs from tear fluid of pSS patients. Following LC-MS of EVs extracted from tear fluid, DAVID analysis (v 6.7, https://david.ncifcrf.gov) was applied using a FDR with a maximum cut-off of 5%. Cellular processes for the upregulated proteins in the pSS patients were identified, and FunRich (http://www.funrich.org/) was then used to visualise the segment of proteins involved. **a** Upregulated signalling pathways distinguished in EVs isolated from tears of pSS patients, as compared to non-SS subjects. **b** Comparing pSS patients to healthy controls, the most upregulated of cellular processes in the pSS patient group was again retina homeostasis, followed by other central innate and adaptive immune responses. Percentage values represent the fraction of overexpressed proteins contributing to the upregulation of each cellular process

The DAVID analysis of the pooled tear sample from 15 non-SS subjects and 10 pSS patients, respectively, revealed upregulated cellular pathways in pSS patients involved in retina homeostasis (83.3%), metabolic processes, regulation of NF-kappaB import, erythrocyte homeostasis, MAP kinase cascade, removal of superoxide radicals, regulation of programmed cell death, natural killer cell cytotoxicity and activation, response to oxidative stress, as well as regulation of cell proliferation and apoptotic processes, in downward order, as determined by the percent values of the upregulated proteins contributing to each cellular process (Fig. 3a). Similarly, the most upregulated cellular processes in the pSS patient group when compared to the healthy controls was retina homeostasis (77.8%), followed by other central innate and adaptive immune responses (Fig. 3b).

When comparing non-SS participants to pSS patients, no significant proteins were found to be significantly upregulated in the patients. However, proteins expressed significantly more in the pSS patient group compared to the healthy controls were erythrocyte band 7 integral membrane protein (STOM), Annexin A4 (ANXA4) and Annexin A11 (ANXA11). STOM is involved in neutrophil degranulation and regulation of viral genome replication, while ANXA4 affects NF-kappaB binding, apoptosis, and interleukin-8 secretion. Finally, ANXA11 regulates MHC class II protein complex binding and phagocytosis.

### Whole saliva and EVs isolated from whole saliva revealed proteins vital for innate MHC class I cellular regulation and T cell activation in pSS patients

The LC-MS conducted on stimulated whole saliva from 15 non-SS sicca subjects, 10 pSS patients and 10 healthy controls aided in the identification of significantly upregulated proteins in the patient group when compared to non-SS sicca subjects and healthy controls, respectively (Additional file 6: Table S3 and Additional file 7: Table S4). Furthermore, significantly upregulated proteins for pSS patients found in EVs of whole saliva were distinguished in a similar manner (Additional file 8: Table S5 and Additional file 9: Table S6). However, the DAVID analysis performed on these highly expressed proteins did not reveal any signalling pathways involving cellular processes to be significantly affected in the patient group.

Considering the number of spectral counts, the three most overexpressed proteins in the pSS patient group when compared to the non-SS sicca participants were peptidyl-prolyl cis-trans isomerase FKBP1A (FKBP1A), CD44 antigen (CD44) and B2MG. The most upregulated of these proteins, FKBP1A, plays a role in T cell activation in FOXP3 expression and regulatory T cell suppression, in addition to promoting tumour growth and progression. Meanwhile, B2MG mediates antigen processing and presentation on MHC class I, and innate immunity. When comparing patients with pSS to healthy controls, proteins Secreted Ly-6/uPAR-related protein 1 (SLUR1), B2MG, and Clusterin (CLUS) were highly expressed in the patient group, in declining order. SLUR1 affects acetylcholine receptor activity, cell migration and proliferation, while CLUS plays modulates NF-kappa-B activity and TNF production (Table 5, Additional file 2: Figure S2).

**Table 5:** Highly upregulated proteins in stimulated whole saliva of pSS patients

Number	Gene	Related protein*	Classification and function**
Non-SS vs. pSS			
1	FKBP1A	Peptidyl-prolyl cis-trans isomerase FKBP1A	T cell activation and proliferation, upregulation of NF-kappa-B signalling
2	CD44	CD44 antigen	FOXP3 expression and regulatory T cell suppression, promotes tumour growth
3	B2MG	Beta-2-microglobulin	Antigen processing and presentation on MHC class I, innate immunity
Controls vs. pSS			
1	SLUR1	Secreted Ly-6/uPAR-related protein 1	Acetylcholine receptor activity, cell migration and proliferation
2	B2MG	Beta-2-microglobulin	Antigen processing and presentation on MHC class I, innate immunity
3	CLUS	Clusterin	Innate immunity, modulates NF-kappa-B activity and TNF production

*The three most upregulated immunological proteins in whole saliva of pSS patients deviating in replicates, i.e. number of individuals (frequency), and spectral counts, as identified by proteomics analysis and Scaffold (v 4.4.6, http://www.proteomesoftware.com/products/scaffold/)

**The classification and functions of the proteins presented were identified using publicly available databases, such as UniProt (http://www.uniprot.org)

Viewing the spectral counts of proteins identified in EVs of whole saliva, the three most upregulated proteins in patients with pSS, as related to non-SS sicca participants, included CD44, Major vault protein (MVP), and Neutrophil gelatinase-associated lipocalin (NGAL), also referred to as LCN2. MVP promotes IFNγ-mediated signalling, MAP kinase activity and neutrophil degranulation, while NGAL is a tumour-associated antigen involved in cell adhesion and innate immunity. Comparing the pSS patient group with healthy controls helped distinguish proteins Ficolin-1 (FCN1), CD44 and ANXA4 as upregulated in the patient group, in descending order. The most changed of these proteins in EVs from whole saliva, FCN1, is a pattern-recognition receptor involved in innate immunity and complement activation (Table 6, Additional file 3: Figure S3).

**Table 6:** Highly upregulated proteins in EVs isolated from stimulated whole saliva of pSS patients

Number	Gene	Related protein*	Classification and function**
Non-SS vs. pSS			
1	CD44	CD44 antigen	FOXP3 expression and regulatory T-cell suppression, promotes tumour growth
2	MVP	Major vault protein	IFNγ-mediated signalling, MAP kinase activity, neutrophil degranulation
3	NGAL	Neutrophil gelatinase-associated lipocalin	Innate immunity, tumour-associated antigen, cell adhesion
Controls vs. pSS			
1	FCN1	Ficolin-1	Pattern-recognition receptor in innate immunity, complement activation
2	CD44	CD44 antigen	FOXP3 expression and regulatory T-cell suppression, promotes tumour growth
3	ANXA4	Annexin A4	NF-kappaB binding, apoptosis, IL-8 secretion

*The three most upregulated immunological proteins in whole saliva of pSS patients deviating in replicates i.e. number of individuals (frequency), and spectral counts, as identified by proteomics analysis and Scaffold (v 4.4.6, http://www.proteomesoftware.com/products/scaffold/)

**The classification and functions of the proteins presented were identified using publicly available databases, such as UniProt (http://www.uniprot.org)

## Discussion

Non-SS sicca subjects represent an interesting group, in relation to pSS, since they display the common symptoms of dry eyes and dry mouth, and may also display mild signs of chronic inflammation in their salivary gland tissue. Still, they serologically are autoantibody negative, and their evaluated salivary gland biopsies usually have a focus score value of 0 or < 1. To date, it remains undetermined whether these discrepancies are a result of an alternate disease trajectory, and these subjects remain understudied within the field of proteomics. By investigating morphological patterns of chronic inflammation in the salivary gland tissue of these non-SS sicca subjects, and studying the proteome of their biological fluids through LC-MS, in combination with size-exclusion chromatographic extraction of EVs, we aimed to delineate the cellular pathways propagating disease. By doing so, we may gain further insight into whether these subjects behave more like pSS patients or healthy controls on glandular and protein levels. Thus, additional biomarkers could also be identified and implemented as potential non-invasive diagnostic tools, encompassing both lacrimal and salivary disease target organs. Together, these findings may aid in increasing diagnostic accuracy when evaluating non-SS subjects and patients with pSS and monitoring disease progression.

In order to assess the level of chronic inflammation in minor salivary gland biopsies of the non-SS subjects included in this study, in relation to patients with pSS, the sections were evaluated for mononuclear cells infiltration, tissue damage and fatty replacement [16, 17]. Interestingly, 27% of these non-SS subjects showed some focal chronic inflammation in their salivary gland tissue, which resulted in a slightly positive focus score of < 1, and fatty infiltration in 40% of the cases (Table 1). Meanwhile, 67% of pSS patients had a positive focus score, where 50% of these biopsies were also GC positive, and 83% also had a positive fatty infiltration score (Table 2). Hence, our histopathological evaluation of minor salivary gland biopsies showed clear implications of chronic inflammation in the target organ of non-SS sicca controls, in the form of focal inflammation, fibrosis and fatty infiltration, although to a lesser degree than pSS patients (Fig. 1).

To delineate cellular pathways involving the upregulated proteins identified with LC-MS in the tear fluid samples from pSS patients, in relation to non-SS sicca controls and healthy individuals, GO and Kyoto Encyclopedia of Genes and Genomes (KEGG) pathway overrepresentation analyses were performed using DAVID. Our results demonstrated pathways in the pSS patients involving inflammatory innate immune responses, such as MHC class I antigen processing and presentation, TNF-mediated signalling and IL-1 mediated signalling. Additional T cell receptor signalling of the adaptive immune response, and apoptotic processes through MAP kinase cascade, were also detected (Fig. 2). Interestingly, similar cellular processes were observed as a result of upregulated proteins in the pSS patients, when compared to both non-SS sicca subjects and healthy individuals. Moreover, the three most upregulated proteins identified in tear fluid of pSS patients were similar, when compared to both non-SS subjects and healthy controls, namely LMO7, HUWE1 and TPD52 (Table 4, Additional file 1: Figure S1). Here, LMO7 and HUWE1 are both involved in the post-translational modification processes of ubiquitination [47, 48], similar to the SS autoantigen Ro52 [49], also known as E3 ubiquitin ligase. Meanwhile, TPD52 plays a central role in adaptive immunity by regulating B cell differentiation [50]. Taken together, these identified cellular pathways and proteins in tear fluid of pSS patients imply the involvement of innate and adaptive immune systems, where non-SS sicca subjects showed similar behavioural tendencies as healthy controls on a protein level.

Our proteomic analysis of EVs extracted from pooled tear samples revealed upregulated cellular pathways in pSS patients, as compared to non-SS sicca subjects, involving retina homeostasis, regulation of metabolic processes, programmed cell death, natural killer cell cytotoxicity and cell proliferation. Correspondingly, the most upregulated of cellular processes in the patient group when compared to the healthy controls was also retina homeostasis, in addition to other adaptive and innate immune responses (Fig. 3). Still, no significant proteins were found to be upregulated in the patients when compared to non-SS sicca subjects. This could be a consequence of pooling the tear samples from individuals in each of the study groups prior to EV extraction, due to the compromised tear production in pSS and non-SS subjects, resulting in loss of insight into variability between the samples. Nonetheless, the most upregulated protein in pSS patients as related to healthy controls, STOM, involves neutrophil degranulation and regulation of viral genome replication [51], suggesting the involvement of viral infection in SS pathogenesis, as previously reported [23]. Meanwhile, ANXA4 and ANXA11 involve innate immune responses and phagocytosis [52, 53], implying interplay between innate and adaptive immunity, both as a consequence of disease pathogenesis and probably also as part of the healing process.

The DAVID analysis performed on highly expressed proteins identified in whole saliva, and EVs separated from whole saliva, did not disclose significantly affected signalling pathways in the pSS patient group. Nevertheless, when considering the number of spectral counts, we managed to identify the three most overexpressed proteins in the whole saliva of pSS patients (Table 5, Additional file 2: Figure S2). When compared to non-SS sicca subjects, the two most upregulated of these proteins in pSS patients, FKBP1A and CD44, play a role in adaptive immunity through T-cell activation and proliferation [54, 55], in addition to promoting tumour growth and progression. Concurrently, B2MG is central in innate immunity by mediating antigen processing and presentation on MHC class I [56]. Comparing patients with pSS to healthy controls, CD44 was again identified, in addition to SLUR1, affecting acetylcholine receptor activity, cell migration and proliferation, and CLUS, playing a key role in innate immunity by modulating NF-kappa-B activity and TNF production [57, 58].

Highly upregulated proteins identified in EVs of whole saliva in patients with pSS, as related to non-SS sicca participants, also included CD44, in addition to MVP, and NGAL (LCN2) (Table 6, Additional file 3: Figure S3). We have previously demonstrated NGAL to be upregulated in the proteome of patients with pSS [29]. Being a protein involved in the activation of neutrophils [59] further strengthens the notion of a role for viral infection in the pathogenesis of SS. Hence, NGAL could be viewed as a potential biomarker for SS, whereby screening for NGAL in whole saliva from patients with pSS could provide additional diagnostic accuracy. Interestingly, NGAL has also been reported as a possible disease biomarker in systemic lupus erythematosus (SLE) [60]. Furthermore, CD44 could also be viewed as a potential salivary biomarker for SS, as it was shown to be highly upregulated in both whole saliva and EVs isolated from whole saliva of pSS patients.

## Conclusions

In conclusion, non-SS sicca subjects may demonstrate chronic inflammation in their glandular tissue, in the form of mild mononuclear cell infiltration, along with sicca oral and ocular symptoms, yet lack the characteristic feature of autoantibody production. However, studying the proteome of their biological fluids through LC-MS, in combination with size-exclusion chromatographic extraction of EVs, revealed upregulated cellular pathways propagating disease, where these non-SS sicca subjects showed tendencies similar to healthy controls rather than to pSS patients. Thus, this analysis confirms that pSS patients displaying focal sialadenitis in the salivary gland with focus score ≥ 1 and/or serum autoantibodies represent a distinct entity with an alternate disease trajectory from non-SS subjects that have focus score values of 0 or < 1 in their glandular tissue. Furthermore, the additional panels of biomarkers identified in this study, such as LMO7, HUWE1, NGAL and CD44, could be implemented in future potential non-invasive diagnostics. Together, these findings may aid in increasing diagnostic accuracy when evaluating non-SS subjects and patients with pSS, and monitoring disease progression. Future follow-up studies are necessary in order to validate these biomarkers in larger pSS cohorts, in addition to studying the role and expression pattern of these cellular components immunologically.

## Additional files

Additional file 1: Figure S1. Upregulated protein expression of potential disease biomarkers identified in tear fluid of pSS patients. Considering the mean number of spectral counts for the proteins detected in the study groups included in our LC-MS analyses, the three most upregulated proteins in pSS patients (black) that are involved in immunological reactions, when compared to both non-SS subjects (grey) and healthy controls (white), were LMO7, HUWE1, and TPD52, in descending order. Statistical significance where *p* < 0.01 is indicated by (*), and *p* < 0.001 is highlighted by (**). (TIFF 10515 kb)

Additional file 2: Figure S2. Upregulation of potential disease biomarkers detected in stimulated whole saliva of pSS patients. In view of the mean number of spectral counts of the proteins identified when performing LC-MS analyses, the three most overexpressed proteins in the pSS patient group (black) when compared to the non-SS sicca participants (grey) were FKBP1A, CD44, and B2MG. Meanwhile, when comparing patients with pSS (black) to healthy controls (white), proteins SLUR1, B2MG, and CLUS were highly expressed in the patient group, in declining order. Statistical significance where *p* < 0.01 is indicated by (*), and *p* < 0.001 is highlighted by (**). (TIFF 11015 kb)

Additional file 3: Figure S3. Overexpression of proteins and potential disease biomarkers found in EVs isolated from stimulated whole saliva in pSS patients. Viewing the mean spectral counts of proteins identified in EVs of whole saliva, the three most upregulated proteins in patients with pSS (black), as related to non-SS sicca participants (grey), included CD44, MVP, and NGAL, also referred to as LCN2. Comparing the pSS patient group (black) with healthy controls (white) helped distinguish proteins FCN1, CD44 and ANXA4 as upregulated in the patient group, in decreasing order. Statistical significance where *p* < 0.01 is indicated by (*). (TIFF 10937 kb)

Additional file 4: Table S1. Upregulated proteins in tear fluid of non-SS subjects vs. pSS patients. (PDF 277 kb)

Additional file 5: Table S2. Upregulated proteins in tear fluid of controls vs. pSS patients. (PDF 262 kb)

Additional file 6: Table S3. Upregulated proteins in whole saliva of non-SS subjects vs. pSS patients. (PDF 177 kb)

Additional file 7: Table S4. Upregulated proteins in whole saliva of controls vs. pSS patients. (PDF 40 kb)

Additional file 8: Table S5. Upregulated proteins in EVs isolated from whole saliva of non-SS subjects vs. pSS patients. (PDF 206 kb)

Additional file 9: Table S6. Upregulated proteins in EVs isolated from whole saliva of controls vs. pSS patients. (PDF 211 kb)

## Data Availability

The datasets generated and/or analysed during the current study are not publicly available due to ethical restrictions enforced by the research and medical institutions under licence for the current study. Data are however available from the authors upon reasonable request and with permission of the Regional Medical Ethical Committee of South-East Norway, the University of Oslo and Oslo University Hospital.

## Abbreviations

ACR: American College of Rheumatology
AECG: American-European Consensus Group
ANXA11: Annexin A11
ANXA4: Annexin A4
B2MG: β-2-Microglobulin
CD44: CD44 antigen
CLUS: Clusterin
DAVID: Database for Annotation, Visualization and Integrated Discovery
EV: Extracellular vesicles
FCN1: Ficolin-1
FDR: False discovery rate
FKBP1: Peptidyl-prolyl cis-trans isomerase FKBP1A
GO: Gene ontology
HUWE1: E3 ubiquitin-protein ligase HUWE1
IL: Interleukin
KEGG: Kyoto Encyclopedia of Genes and Genomes
LC-MS: Liquid chromatography-mass spectrometry
LCN2: Neutrophil gelatinase-associated lipocalin
LMO7: LIM domain only protein 7
LSP1: Lymphocyte-specific protein 1 MFI: Median fluorescence intensity
MVP: Major vault protein
NGAL: Neutrophil gelatinase-associated lipocalin
PBS: Phosphate-buffered saline
pSS: Primary Sjögren’s syndrome
S/N: Signal to noise ratio
SLE: Systemic lupus erythematosus
SLUR1: Secreted Ly-6/uPAR-related protein 1
STOM: Erythrocyte band 7 integral membrane protein
TIC: Total ion current
TNF: Tumour necrosis factor
TPD52: Tumour protein D52
