# Supplementary figures and images for: Proteomic and histopathological characterisation of sicca subjects and primary Sjögren’s syndrome patients reveals promising tear, saliva and extracellular vesicle disease biomarkers
Source: Arthritis Res Ther. 2019 Jul 31;21:181. doi: 10.1186/s13075-019-1961-4 (PMC6670195; doi:10.1186/s13075-019-1961-4)

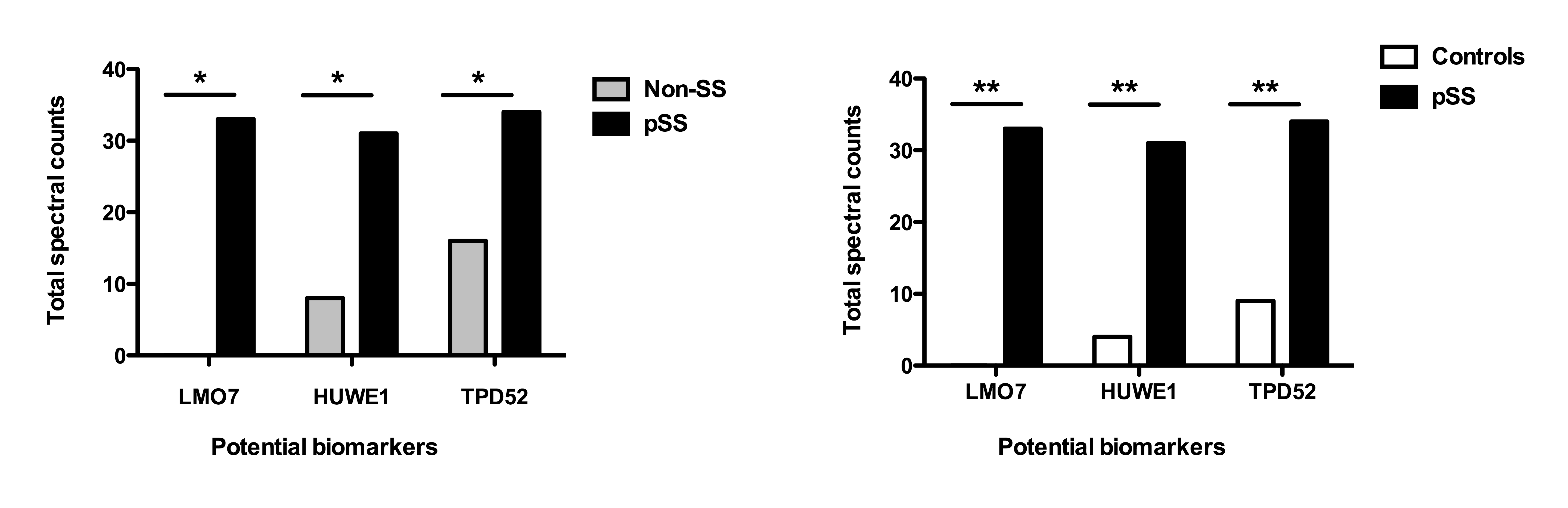

Supplement: Supplementary file 1 — Figure S1. Upregulated protein expression of potential disease biomarkers identified in tear fluid of pSS patients. Considering the mean number of spectral counts for the proteins detected in the study groups included in our LC-MS analyses, the three most upregulated proteins in pSS patients (black) that are involved in immunological reactions, when compared to both non-SS subjects (grey) and healthy controls (white), were LMO7, HUWE1, and TPD52, in descending order. Statistical significance where p < 0.01 is indicated by (*), and p < 0.001 is highlighted by (**). (TIFF 10515 kb) [file 13075_2019_1961_MOESM1_ESM.tiff]

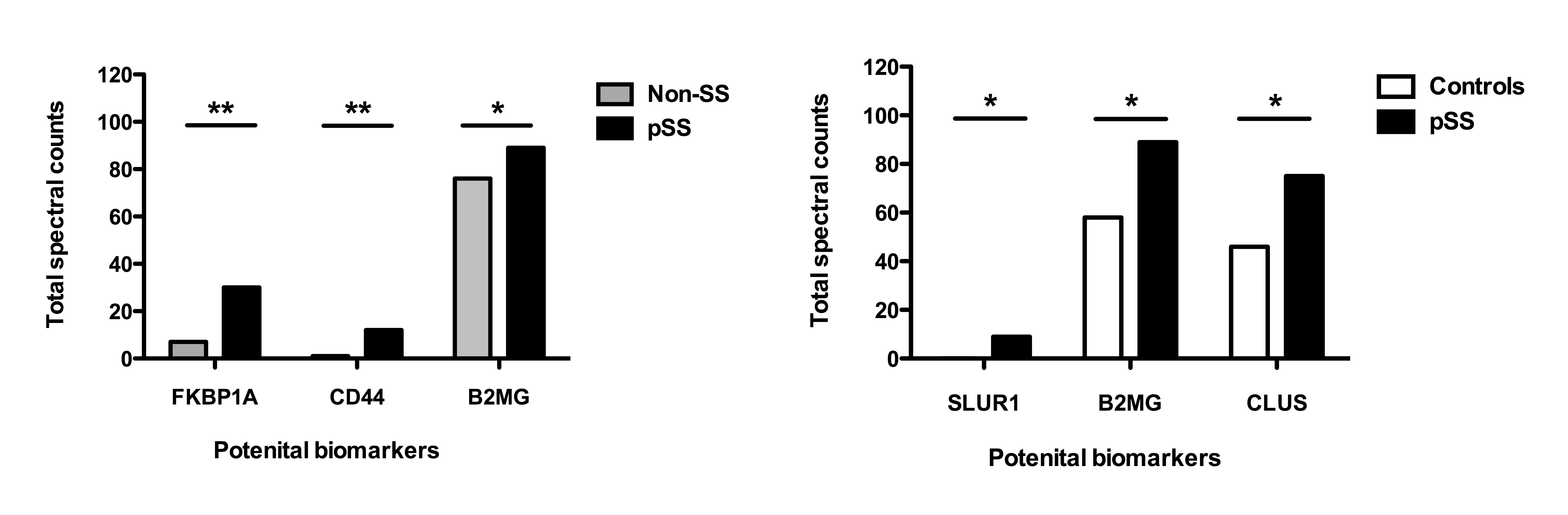

Supplement: Supplementary file 2 — Figure S2. Upregulation of potential disease biomarkers detected in stimulated whole saliva of pSS patients. In view of the mean number of spectral counts of the proteins identified when performing LC-MS analyses, the three most overexpressed proteins in the pSS patient group (black) when compared to the non-SS sicca participants (grey) were FKBP1A, CD44, and B2MG. Meanwhile, when comparing patients with pSS (black) to healthy controls (white), proteins SLUR1, B2MG, and CLUS were highly expressed in the patient group, in declining order. Statistical significance where p < 0.01 is indicated by (*), and p < 0.001 is highlighted by (**). (TIFF 11015 kb) [file 13075_2019_1961_MOESM2_ESM.tiff]

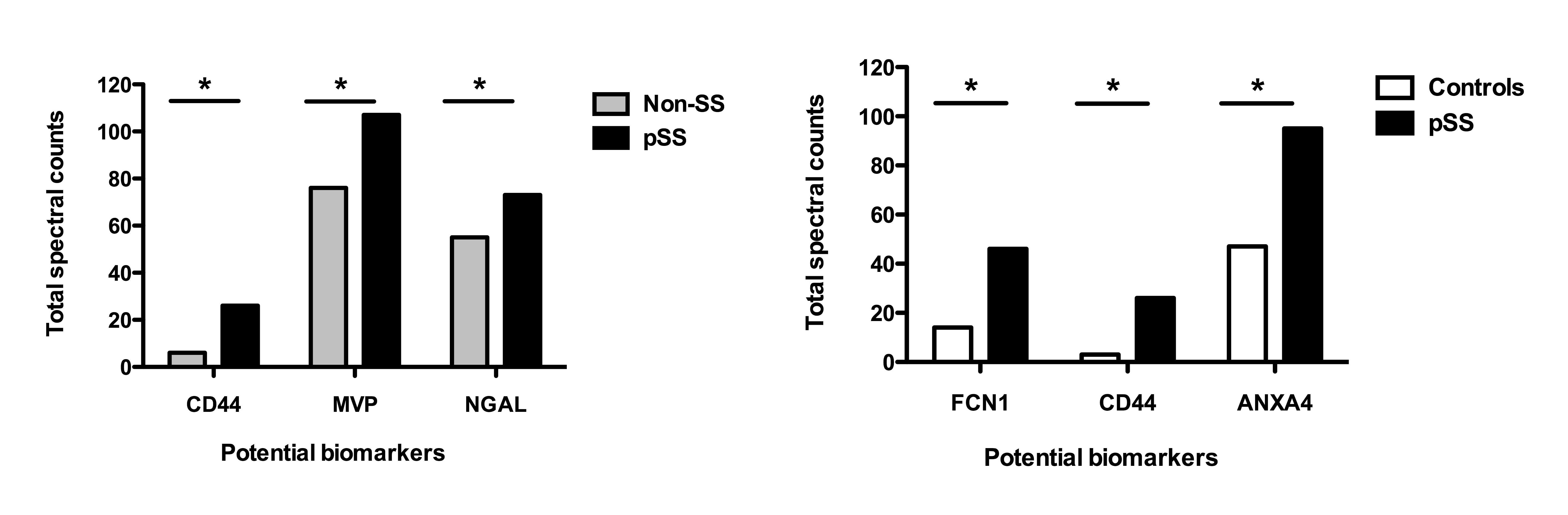

Supplement: Supplementary file 3 — Figure S3. Overexpression of proteins and potential disease biomarkers found in EVs isolated from stimulated whole saliva in pSS patients. Viewing the mean spectral counts of proteins identified in EVs of whole saliva, the three most upregulated proteins in patients with pSS (black), as related to non-SS sicca participants (grey), included CD44, MVP, and NGAL, also referred to as LCN2. Comparing the pSS patient group (black) with healthy controls (white) helped distinguish proteins FCN1, CD44 and ANXA4 as upregulated in the patient group, in decreasing order. Statistical significance where p < 0.01 is indicated by (*). (TIFF 10937 kb) [file 13075_2019_1961_MOESM3_ESM.tiff]
