# Supplementary material for: Proteomic and histopathological characterisation of sicca subjects and primary Sjögren’s syndrome patients reveals promising tear, saliva and extracellular vesicle disease biomarkers
Source: Arthritis Res Ther. 2019 Jul 31;21:181. doi: 10.1186/s13075-019-1961-4 (PMC6670195; doi:10.1186/s13075-019-1961-4)
Supplement: Supplementary file 4 — Table S1. Upregulated proteins in tear fluid of non-SS subjects vs. pSS patients. (PDF 277 kb) [file 13075_2019_1961_MOESM4_ESM.pdf]

Table S1. Upregulated proteins in tear fluid of non-SS subjects vs. pSS patients

Gene name	T-Test (P-Value)	SC non-SS	SC pSS
TCPH_HUMAN	< 0.00010	7	43
CN166_HUMAN	< 0.00010	0	28
EVPL_HUMAN	< 0.00010	1	75
PEPL_HUMAN	< 0.00010	26	131
EIF3C_HUMAN	< 0.00010	2	27
ECHM_HUMAN	< 0.00010	0	20
EIF3L_HUMAN	< 0.00010	0	23
CCDC6_HUMAN	< 0.00010	0	24
HNRPM_HUMAN	< 0.00010	9	54
TKFC_HUMAN	< 0.00010	18	62
SPTB2_HUMAN	< 0.00010	6	71
RAB25_HUMAN	< 0.00010	4	23
RRBP1_HUMAN	< 0.00010	0	33
LASP1_HUMAN	< 0.00010	4	29
UGPA_HUMAN	< 0.00010	81	128
PSDE_HUMAN	< 0.00010	2	28
VPS35_HUMAN	< 0.00010	6	51
DHX9_HUMAN	< 0.00010	0	29
ZN185_HUMAN	< 0.00010	5	51
MPI_HUMAN	< 0.00010	0	20
GIPC1_HUMAN	< 0.00010	2	22
LMO7_HUMAN	< 0.00010	0	33
HNRPF_HUMAN	< 0.00010	4	27
ILF3_HUMAN	< 0.00010	2	33
YBOX1_HUMAN	< 0.00010	0	15
NSF_HUMAN	< 0.00010	19	59
DDAH2_HUMAN	< 0.00010	0	25
SCEL_HUMAN	< 0.00010	7	59
AHNK_HUMAN	< 0.00010	91	283
DDX3X_HUMAN	< 0.00010	0	22
PSMD5_HUMAN	< 0.00010	0	17
ILF2_HUMAN	< 0.00010	6	36
PSMD4_HUMAN	< 0.00010	0	21
OXSR1_HUMAN	< 0.00010	4	38
CIP4_HUMAN	< 0.00010	2	26
ACON_HUMAN	< 0.00010	4	28
SYEP_HUMAN	< 0.00010	0	15
PRS7_HUMAN	< 0.00010	7	39
ANT3_HUMAN	< 0.00010	3	56
TCPE_HUMAN	< 0.00010	16	49
GRP75_HUMAN	< 0.00010	1	26
ICAL_HUMAN	< 0.00010	67	121
HXK1_HUMAN	< 0.00010	14	68
DYHC1_HUMAN	< 0.00010	57	143
DYN2_HUMAN	< 0.00010	0	27
ROA1_HUMAN	< 0.00010	36	70
RL13_HUMAN	< 0.00010	5	26

KINH_HUMAN	< 0.00010	5	28
S12A2_HUMAN	< 0.00010	16	52
DDX1_HUMAN	< 0.00010	4	27
HUWE1_HUMAN	< 0.00010	8	31
FIBA_HUMAN	< 0.00010	143	206
ZO2_HUMAN	< 0.00010	1	17
BCAS1_HUMAN	< 0.00010	9	35
HPRT_HUMAN	< 0.00010	6	28
CASP7_HUMAN	< 0.00010	0	19
KS6A3_HUMAN	< 0.00010	4	32
XRCC5_HUMAN	< 0.00010	0	31
RL18_HUMAN	< 0.00010	0	15
CTNA1_HUMAN	< 0.00010	0	32
TWF1_HUMAN	< 0.00010	23	47
LMNA_HUMAN	< 0.00010	32	90
DCTN1_HUMAN	< 0.00010	7	31
DD19A_HUMAN	< 0.00010	3	21
SYQ_HUMAN	< 0.00010	9	42
EIF3I_HUMAN	< 0.00010	2	19
PSD11_HUMAN	< 0.00010	20	42
FLNB_HUMAN	< 0.00010	73	148
PIN1_HUMAN	< 0.00010	0	16
NEDD8_HUMAN	< 0.00010	0	23
PRS8_HUMAN	< 0.00010	7	24
RS7_HUMAN	< 0.00010	1	20
USO1_HUMAN	< 0.00010	4	33
MX1_HUMAN	< 0.00010	17	72
TYPH_HUMAN	< 0.00010	207	292
GBP1_HUMAN	0,0001	10	47
S100P_HUMAN	0,0001	30	79
CYFP1_HUMAN	0,00011	3	27
LRC47_HUMAN	0,00012	13	37
SH24A_HUMAN	0,00012	1	18
NXP20_HUMAN	0,00013	0	18
DHSO_HUMAN	0,00013	71	115
IF16_HUMAN	0,00014	0	22
MVP_HUMAN	0,00014	122	168
ANXA9_HUMAN	0,00014	0	24
SHLB2_HUMAN	0,00014	5	23
SPTN1_HUMAN	0,00015	33	106
5NT3A_HUMAN	0,00016	0	12
TCPD_HUMAN	0,00017	32	70
ES8L2_HUMAN	0,00019	0	26
TERA_HUMAN	0,00019	186	248
DSG2_HUMAN	0,0002	6	29
LRBA_HUMAN	0,0002	2	20
UBA7_HUMAN	0,00021	31	67
S10A9_HUMAN	0,00025	70	121
EHD1_HUMAN	0,00026	1	23
TCPZ_HUMAN	0,00028	26	50

INVO_HUMAN	0,00028	18	74
LRRF1_HUMAN	0,00029	0	13
SYDC_HUMAN	0,0003	8	33
GFPT1_HUMAN	0,00032	58	96
FLII_HUMAN	0,00032	1	20
GBP2_HUMAN	0,00033	3	30
ALDOC_HUMAN	0,00033	55	81
PFD2_HUMAN	0,00033	0	12
HDHD2_HUMAN	0,00034	0	12
TPD52_HUMAN	0,00035	16	34
SNX2_HUMAN	0,00039	6	31
ROA2_HUMAN	0,00039	95	135
AATC_HUMAN	0,00043	2	23
PARK7_HUMAN	0,00044	56	84
EPIPL_HUMAN	0,00045	20	68
FA49B_HUMAN	0,00047	13	38
NUDT5_HUMAN	0,00047	10	28
DIAP1_HUMAN	0,00047	0	16
RS9_HUMAN	0,00051	0	17
TCPA_HUMAN	0,00052	46	79
MAOX_HUMAN	0,00052	0	13
RL7_HUMAN	0,00056	0	15
DDX17_HUMAN	0,00059	0	21
PSD13_HUMAN	0,00061	8	22
GCN1L_HUMAN	0,00062	0	10
TPD54_HUMAN	0,00066	17	35
GALK1_HUMAN	0,00071	0	11
SYWC_HUMAN	0,00072	65	119
RUVB1_HUMAN	0,00074	0	16
SHOT1_HUMAN	0,00075	0	16
AMBP_HUMAN	0,00082	1	15
HP1B3_HUMAN	0,00083	3	21
BAX_HUMAN	0,00091	0	9
TKT_HUMAN	0,00097	118	152
LDHB_HUMAN	0,001	40	63
TPM4_HUMAN	0,001	88	115
MDHM_HUMAN	0,001	57	76
PLEC_HUMAN	0,0011	17	69
MYO6_HUMAN	0,0011	3	16
AL7A1_HUMAN	0,0012	40	62
IPO7_HUMAN	0,0012	2	15
COPG1_HUMAN	0,0013	15	43
MK01_HUMAN	0,0013	4	23
FAS_HUMAN	0,0013	12	52
IF2G_HUMAN	0,0013	12	25
VATE1_HUMAN	0,0013	0	14
TB182_HUMAN	0,0013	0	9
ST134_HUMAN	0,0014	0	19
UBA6_HUMAN	0,0014	0	15
AIMP1_HUMAN	0,0014	4	16

PP1B_HUMAN	0,0014	9	31
XPO1_HUMAN	0,0014	1	17
SYVC_HUMAN	0,0015	1	16
HNRPU_HUMAN	0,0015	19	35
EIF3A_HUMAN	0,0016	7	27
RCC1_HUMAN	0,0016	0	15
AP1B1_HUMAN	0,0017	11	41
EFHD2_HUMAN	0,0017	6	19
TRI29_HUMAN	0,0017	0	16
VP13C_HUMAN	0,0018	0	12
A2AP_HUMAN	0,0018	4	27
NIBL1_HUMAN	0,0018	67	100
DEOC_HUMAN	0,0018	0	10
RLA2_HUMAN	0,0019	24	44
AXA81_HUMAN	0,0019	12	31
ROA3_HUMAN	0,002	12	36
PRS4_HUMAN	0,0021	0	15
IMDH2_HUMAN	0,0021	1	11
GNAO_HUMAN	0,0021	0	13
XDH_HUMAN	0,0021	12	44
KCRU_HUMAN	0,0022	0	13
RL6_HUMAN	0,0022	2	16
WASF2_HUMAN	0,0023	0	10
CRYM_HUMAN	0,0024	4	19
PGM2_HUMAN	0,0024	17	49
SH3L2_HUMAN	0,0024	3	17
TNAP2_HUMAN	0,0025	3	16
METK2_HUMAN	0,0025	10	24
BAG1_HUMAN	0,0025	0	14
ECH1_HUMAN	0,0025	0	9
ATPA_HUMAN	0,0026	56	84
ANXA6_HUMAN	0,0026	0	17
DDX6_HUMAN	0,0027	0	9
SYG_HUMAN	0,0027	2	15
IMPA1_HUMAN	0,0027	22	38
OPLA_HUMAN	0,0028	0	12
PAK2_HUMAN	0,0029	0	8
B2MG_HUMAN	0,003	11	23
TLN1_HUMAN	0,0031	80	119
THIO_HUMAN	0,0031	41	55
PRS6B_HUMAN	0,0031	0	11
EIF3B_HUMAN	0,0032	1	15
RTCB_HUMAN	0,0032	0	12
ARHL2_HUMAN	0,0035	11	25
SYK_HUMAN	0,0035	6	26
DDB1_HUMAN	0,0036	0	13
PDCD4_HUMAN	0,0036	17	30
TCTP_HUMAN	0,0037	3	16
ULA1_HUMAN	0,0037	0	9
PPCS_HUMAN	0,0037	4	17

DC1L2_HUMAN	0,0038	1	9	
1433E_HUMAN	0,0038	198	234	
COPA_HUMAN	0,0038	8	25	
ML12A_HUMAN	0,0038	43	65	
EIF3D_HUMAN	0,004	2	12	
CPNS2_HUMAN	0,004	0	11	
MYH14_HUMAN	0,0041	753	853	
GMPPB_HUMAN	0,0042	5	18	
SLK_HUMAN	0,0043	10	21	
DX39B_HUMAN	0,0044	13	36	
ERF1_HUMAN	0,0046	2	15	
SYCC_HUMAN	0,0047	0	8	
CALL5_HUMAN	0,0048	3	17	
PCY2_HUMAN	0,005	0	7	
KGUA_HUMAN	0,0052	0	9	
PLIN3_HUMAN	0,0053	92	117	
PRS6A_HUMAN	0,0053	15	33	
OSBP1_HUMAN	0,0054	4	16	
VIGLN_HUMAN	0,0054	5	21	
SC22B_HUMAN	0,0055	1	12	
IGHM_HUMAN	0,0055	25	64	
FUBP2_HUMAN	0,0056	3	16	
CALL3_HUMAN	0,0057	34	44	
UBP5_HUMAN	0,0058	58	73	
GLRX1_HUMAN	0,0058	16	28	
PDC6I_HUMAN	0,0058	112	134	
LIMA1_HUMAN	0,0062	0	12	
PPIA_HUMAN	0,0064	159	184	
GPX1_HUMAN	0,0064	46	58	
HDDC2_HUMAN	0,0065	1	10	
AT1A1_HUMAN	0,0066	0	8	
SRP14_HUMAN	0,0066	0	7	
IL1RA_HUMAN	0,0067	71	85	
ERF3A_HUMAN	0,0069	14	31	
GSDMB_HUMAN	0,007	0	11	
BDH2_HUMAN	0,0073	23	42	
ECM1_HUMAN	0,0073	0	7	
GSTT1_HUMAN	0,0076	3	22	
VATB2_HUMAN	0,0076	19	35	
TIF1B_HUMAN	0,0078	0	9	
PDC10_HUMAN	0,0079	0	8	
CHM4A_HUMAN	0,0081	0	8	
PRKDC_HUMAN	0,0082	1	9	
SC23B_HUMAN	0,0084	0	8	
DHE3_HUMAN	0,0085	2	19	
IFIT1_HUMAN	0,0089	0	9	
LAP2A_HUMAN	0,0089	0	11	
ST2B1_HUMAN	0,0091	0	8	
MAT2B_HUMAN	0,0092	5	15	
SYFB_HUMAN	0,0092	0	11	

TOM1_HUMAN	0,0094	6	20
NIF3L_HUMAN	0,0095	0	7
PTN6_HUMAN	0,0095	1	9
SYYC_HUMAN	0,0095	0	8
TSTD1_HUMAN	0,0096	4	16
IDHC_HUMAN	0,0096	153	177
PHYD1_HUMAN	0,0097	12	22
HEBP1_HUMAN	0,0097	1	9
VTDB_HUMAN	0,0098	18	35
USP9X_HUMAN	0,0098	0	7
SHLB1_HUMAN	0,0098	0	9
MARK2_HUMAN	0,0099	0	7
CIRBP_HUMAN	0,01	8	21
ACLY_HUMAN	0,01	2	14
CDK6_HUMAN	0,01	0	7
SAHH3_HUMAN	0,011	0	11
RL7A_HUMAN	0,011	5	17
RN213_HUMAN	0,011	0	7
ANXA4_HUMAN	0,011	49	76
VMA5A_HUMAN	0,012	4	20
IC1_HUMAN	0,012	6	28
IMB1_HUMAN	0,012	51	82
MARCS_HUMAN	0,012	0	9
TCPQ_HUMAN	0,012	73	89
CUL3_HUMAN	0,013	0	7
SYMC_HUMAN	0,013	0	7
RASL1_HUMAN	0,013	0	8
DNJB1_HUMAN	0,013	0	5
SPRR3_HUMAN	0,013	8	29
KYNU_HUMAN	0,014	62	83
