# Supplementary material for: Proteomic and histopathological characterisation of sicca subjects and primary Sjögren’s syndrome patients reveals promising tear, saliva and extracellular vesicle disease biomarkers
Source: Arthritis Res Ther. 2019 Jul 31;21:181. doi: 10.1186/s13075-019-1961-4 (PMC6670195; doi:10.1186/s13075-019-1961-4)
Supplement: Supplementary file 5 — Table S2. Upregulated proteins in tear fluid of controls vs. pSS patients. (PDF 262 kb) [file 13075_2019_1961_MOESM5_ESM.pdf]

Table S2. Upregulated proteins in tear fluid of controls vs. pSS patients

Gene name	T-Test (P-Value)	SC controls	SC pSS
TCPH_HUMAN	< 0.00010	2	43
RAB25_HUMAN	< 0.00010	0	23
ACON_HUMAN	< 0.00010	1	28
LASP1_HUMAN	< 0.00010	0	29
SYRC_HUMAN	< 0.00010	6	50
COPG1_HUMAN	< 0.00010	2	43
UBA7_HUMAN	< 0.00010	11	67
EIF3C_HUMAN	< 0.00010	1	27
S12A2_HUMAN	< 0.00010	4	52
PSD11_HUMAN	< 0.00010	6	42
TKFC_HUMAN	< 0.00010	8	62
CN166_HUMAN	< 0.00010	0	28
VPS35_HUMAN	< 0.00010	1	51
NUDT5_HUMAN	< 0.00010	2	28
IF2G_HUMAN	< 0.00010	2	25
HPRT_HUMAN	< 0.00010	1	28
HNRPM_HUMAN	< 0.00010	4	54
PRS8_HUMAN	< 0.00010	3	24
NSF_HUMAN	< 0.00010	9	59
SPTB2_HUMAN	< 0.00010	0	71
ILF2_HUMAN	< 0.00010	2	36
EVPL_HUMAN	< 0.00010	2	75
AHNK_HUMAN	< 0.00010	31	283
FLNB_HUMAN	< 0.00010	33	148
DYHC1_HUMAN	< 0.00010	20	143
ZN185_HUMAN	< 0.00010	2	51
CCDC6_HUMAN	< 0.00010	0	24
PSD13_HUMAN	< 0.00010	2	22
HNRPU_HUMAN	< 0.00010	5	35
TCPA_HUMAN	< 0.00010	24	79
ILF3_HUMAN	< 0.00010	0	33
RRBP1_HUMAN	< 0.00010	0	33
SYQ_HUMAN	< 0.00010	0	42
PEPL_HUMAN	< 0.00010	22	131
DDX1_HUMAN	< 0.00010	1	27
PRS7_HUMAN	< 0.00010	4	39
HXK1_HUMAN	< 0.00010	5	68
ICAL_HUMAN	< 0.00010	31	121
DHX9_HUMAN	< 0.00010	0	29
SYDC_HUMAN	< 0.00010	1	33
DPYD_HUMAN	< 0.00010	8	56
MPI_HUMAN	< 0.00010	0	20
DSG2_HUMAN	< 0.00010	1	29
LMO7_HUMAN	< 0.00010	0	33
AP2A2_HUMAN	< 0.00010	0	24
YBOX1_HUMAN	< 0.00010	0	15
OTUB1_HUMAN	< 0.00010	34	83

HUWE1_HUMAN	< 0.00010	4	31
DDAH2_HUMAN	< 0.00010	0	25
TWF1_HUMAN	< 0.00010	13	47
PSDE_HUMAN	< 0.00010	3	28
KS6A3_HUMAN	< 0.00010	1	32
ANT3_HUMAN	< 0.00010	2	56
DDX3X_HUMAN	< 0.00010	0	22
PSMD5_HUMAN	< 0.00010	0	17
GRP75_HUMAN	< 0.00010	0	26
PSMD4_HUMAN	< 0.00010	0	21
GPD1L_HUMAN	< 0.00010	1	23
CYFP1_HUMAN	< 0.00010	0	27
LDHB_HUMAN	< 0.00010	17	63
MVP_HUMAN	< 0.00010	69	168
ZO2_HUMAN	< 0.00010	0	17
SYEP_HUMAN	< 0.00010	0	15
PARK7_HUMAN	< 0.00010	29	84
MX1_HUMAN	< 0.00010	5	72
NIBL1_HUMAN	< 0.00010	29	100
BCAS1_HUMAN	0,0001	10	35
C1TC_HUMAN	0,00013	12	50
PLSI_HUMAN	0,00013	14	66
ECHM_HUMAN	0,00013	2	20
ROA1_HUMAN	0,00014	21	70
AL1L1_HUMAN	0,00014	45	134
OXSR1_HUMAN	0,00015	4	38
TPD52_HUMAN	0,00017	9	34
DD19A_HUMAN	0,00018	2	21
RHG01_HUMAN	0,00018	0	26
LRC47_HUMAN	0,0002	6	37
MAT2B_HUMAN	0,00024	0	15
LRBA_HUMAN	0,00025	0	20
AP1B1_HUMAN	0,00026	2	41
SCEL_HUMAN	0,00027	9	59
SPTN1_HUMAN	0,00027	14	106
TERA_HUMAN	0,00028	114	248
FIBA_HUMAN	0,00031	83	206
DYN2_HUMAN	0,00033	1	27
ARL3_HUMAN	0,00036	3	20
EFHD2_HUMAN	0,00036	2	19
ROA2_HUMAN	0,00037	51	135
HNRPF_HUMAN	0,00038	4	27
RS7_HUMAN	0,00039	0	20
RL18_HUMAN	0,00042	0	15
CTNA1_HUMAN	0,00042	0	32
BDH2_HUMAN	0,00043	10	42
TCPD_HUMAN	0,00043	20	70
HP1B3_HUMAN	0,00046	0	21
ALDOC_HUMAN	0,00049	26	81
SH24A_HUMAN	0,00049	0	18

EIF3A_HUMAN	0,00049	2	27
IGHM_HUMAN	0,0005	12	64
ERF3A_HUMAN	0,00054	4	31
XRCC5_HUMAN	0,00057	1	31
USO1_HUMAN	0,00058	3	33
PP1B_HUMAN	0,00058	0	31
TRI16_HUMAN	0,00072	3	22
PIN1_HUMAN	0,00072	0	16
NEDD8_HUMAN	0,00074	0	23
EMAL4_HUMAN	0,00075	1	13
VIGLN_HUMAN	0,00077	0	21
IMPA1_HUMAN	0,0008	9	38
TNAP2_HUMAN	0,0008	0	16
PSMD3_HUMAN	0,00081	9	32
UGPA_HUMAN	0,00083	55	128
TCPE_HUMAN	0,00095	14	49
TYPH_HUMAN	0,00097	138	292
SLK_HUMAN	0,00099	4	21
RL13_HUMAN	0,001	6	26
E41L1_HUMAN	0,001	9	47
KINH_HUMAN	0,0011	6	28
IPO7_HUMAN	0,0011	0	15
EHD1_HUMAN	0,0011	0	23
IF16_HUMAN	0,0012	0	22
ANXA9_HUMAN	0,0012	0	24
EIF3I_HUMAN	0,0012	2	19
NUCL_HUMAN	0,0012	22	70
GFPT1_HUMAN	0,0012	36	96
COPA_HUMAN	0,0013	3	25
MDHM_HUMAN	0,0013	28	76
IQGA1_HUMAN	0,0013	122	262
GLRX1_HUMAN	0,0014	6	28
PSMD2_HUMAN	0,0015	23	68
MYH14_HUMAN	0,0016	430	853
AIMP1_HUMAN	0,0016	2	16
RL7A_HUMAN	0,0016	0	17
FUBP2_HUMAN	0,0016	0	16
TLN1_HUMAN	0,0017	42	119
PLEC_HUMAN	0,0018	6	69
HNRPQ_HUMAN	0,002	4	19
LRRF1_HUMAN	0,002	0	13
RABP2_HUMAN	0,002	3	22
SH3L2_HUMAN	0,0021	0	17
RL6_HUMAN	0,0021	0	16
CIRBP_HUMAN	0,0022	3	21
COR1B_HUMAN	0,0022	17	53
IC1_HUMAN	0,0022	2	28
PFD2_HUMAN	0,0023	0	12
LMNA_HUMAN	0,0023	24	90
PLAK_HUMAN	0,0023	6	31

KYNU_HUMAN	0,0023	34	83
METK2_HUMAN	0,0024	5	24
ACY1_HUMAN	0,0025	0	15
GBP2_HUMAN	0,0027	3	30
SYK_HUMAN	0,0027	2	26
ENPL_HUMAN	0,0027	24	72
TCPZ_HUMAN	0,0028	19	50
UBE2N_HUMAN	0,0028	8	31
DCTN1_HUMAN	0,0029	5	31
SNX2_HUMAN	0,0029	3	31
ASC_HUMAN	0,0029	2	17
DIAP1_HUMAN	0,003	0	16
SHLB2_HUMAN	0,0031	5	23
RS9_HUMAN	0,0031	0	17
EIF3L_HUMAN	0,0032	5	23
PSA1_HUMAN	0,0033	9	41
RL7_HUMAN	0,0034	0	15
ATPA_HUMAN	0,0035	36	84
RL11_HUMAN	0,0035	0	17
DDX17_HUMAN	0,0035	0	21
ES8L2_HUMAN	0,0036	2	26
RAB7A_HUMAN	0,0036	16	43
PLIN3_HUMAN	0,0036	51	117
GCN1L_HUMAN	0,0036	0	10
OSBP1_HUMAN	0,0037	1	16
TCPQ_HUMAN	0,0037	37	89
5NT3A_HUMAN	0,0037	1	12
IMDH2_HUMAN	0,0038	0	11
DX39B_HUMAN	0,0038	6	36
ERF1_HUMAN	0,004	0	15
GALK1_HUMAN	0,0041	0	11
RUVB1_HUMAN	0,0042	0	16
ENSA_HUMAN	0,0042	0	12
SYVC_HUMAN	0,0042	0	16
PA2G4_HUMAN	0,0043	6	30
RS14_HUMAN	0,0046	4	18
1433E_HUMAN	0,0046	125	234
CASP7_HUMAN	0,0049	2	19
BAX_HUMAN	0,0049	0	9
PDCD4_HUMAN	0,0051	9	30
TPD54_HUMAN	0,0054	13	35
MYO6_HUMAN	0,0056	2	16
NT5C_HUMAN	0,0058	0	18
HINT1_HUMAN	0,0061	8	35
RS13_HUMAN	0,0062	0	10
PGM2_HUMAN	0,0062	9	49
DHSO_HUMAN	0,0063	49	115
CIP4_HUMAN	0,0063	4	26
XDH_HUMAN	0,0065	8	44
VATE1_HUMAN	0,0065	0	14

TB182_HUMAN	0,0066	0	9
PNPH_HUMAN	0,0066	0	17
ST134_HUMAN	0,0066	0	19
UBA6_HUMAN	0,0066	0	15
RLA2_HUMAN	0,0067	15	44
SC31A_HUMAN	0,0071	5	30
GPX1_HUMAN	0,0071	28	58
RCC1_HUMAN	0,0074	0	15
ROA3_HUMAN	0,0077	9	36
HDHD2_HUMAN	0,0078	1	12
TRI29_HUMAN	0,0079	0	16
LGUL_HUMAN	0,0079	1	14
SET_HUMAN	0,0079	19	51
NXP20_HUMAN	0,0079	2	18
SPRR3_HUMAN	0,008	5	29
VP13C_HUMAN	0,008	0	12
S10A9_HUMAN	0,0081	49	121
VAT1_HUMAN	0,0082	2	16
DEOC_HUMAN	0,0082	0	10
S100P_HUMAN	0,0086	25	79
PRS4_HUMAN	0,009	0	15
SHOT1_HUMAN	0,0091	1	16
HSP74_HUMAN	0,0091	27	68
GNAO_HUMAN	0,0091	0	13
EPIPL_HUMAN	0,0093	16	68
KCRU_HUMAN	0,0094	0	13
WASF2_HUMAN	0,0099	0	10
PVRL4_HUMAN	0,01	0	6
HDDC2_HUMAN	0,01	0	10
PRKDC_HUMAN	0,01	0	9
ECH1_HUMAN	0,011	0	9
ANXA6_HUMAN	0,011	0	17
PDIA4_HUMAN	0,011	13	41
VMA5A_HUMAN	0,011	1	20
DDX6_HUMAN	0,011	0	9
MAOX_HUMAN	0,011	1	13
FLII_HUMAN	0,011	3	20
DCTN2_HUMAN	0,011	26	68
OPLA_HUMAN	0,011	0	12
PAK2_HUMAN	0,012	0	8
SC22B_HUMAN	0,012	0	12
