# Supplementary material for: Proteomic and histopathological characterisation of sicca subjects and primary Sjögren’s syndrome patients reveals promising tear, saliva and extracellular vesicle disease biomarkers
Source: Arthritis Res Ther. 2019 Jul 31;21:181. doi: 10.1186/s13075-019-1961-4 (PMC6670195; doi:10.1186/s13075-019-1961-4)
Supplement: Supplementary file 6 — Table S3. Upregulated proteins in whole saliva of non-SS subjects vs. pSS patients. (PDF 177 kb) [file 13075_2019_1961_MOESM6_ESM.pdf]

Table S3. Upregulated proteins in whole saliva of non-SS subjects vs. pSS patients

Gene name	T-Test (P-Value)	SC non-SS	SC pSS
FKB1A_HUMAN	< 0,00010	7	30
CH10_HUMAN	0,00035	4	18
CD44_HUMAN	0,0007	1	12
LAC2_HUMAN	0,001	436	606
KV303_HUMAN	0,0022	22	55
B2MG_HUMAN	0,0045	76	89
GLU2B_HUMAN	0,0047	25	32
IGHG1_HUMAN	0,0076	880	997
KV304_HUMAN	0,011	0	53
WDR1_HUMAN	0,014	109	127
B4GT1_HUMAN	0,018	4	12
PSA5_HUMAN	0,027	1	7
LV105_HUMAN	0,032	0	8
GBB2_HUMAN	0,033	23	42
DSC3_HUMAN	0,033	3	15
ARP3_HUMAN	0,035	123	145
