# Supplementary material for: Proteomic and histopathological characterisation of sicca subjects and primary Sjögren’s syndrome patients reveals promising tear, saliva and extracellular vesicle disease biomarkers
Source: Arthritis Res Ther. 2019 Jul 31;21:181. doi: 10.1186/s13075-019-1961-4 (PMC6670195; doi:10.1186/s13075-019-1961-4)
Supplement: Supplementary file 7 — Table S4. Upregulated proteins in whole saliva of controls vs. pSS patients. (PDF 40 kb) [file 13075_2019_1961_MOESM7_ESM.pdf]

Table S4. Upregulated proteins in whole saliva of controls vs. pSS patients

Gene name	T-Test (P-Value)	SC controls	SC pSS
CH10_HUMAN	0,0065	5	18
ACPH_HUMAN	0,0066	0	16
SLUR1_HUMAN	0,01	0	9
ASAH1_HUMAN	0,012	6	20
CFAB_HUMAN	0,013	55	106
TM11D_HUMAN	0,013	49	76
CATS_HUMAN	0,02	5	23
IGHG1_HUMAN	0,024	630	997
KV303_HUMAN	0,024	23	55
B2MG_HUMAN	0,025	58	89
GDIR2_HUMAN	0,029	87	129
PRB2_HUMAN	0,029	50	85
CLUS_HUMAN	0,031	46	75
KV304_HUMAN	0,032	0	53
RET4_HUMAN	0,034	18	37
1433F_HUMAN	0,034	9	43
UTER_HUMAN	0,037	0	13
NHRF1_HUMAN	0,048	0	10
