# Supplementary material for: Proteomic and histopathological characterisation of sicca subjects and primary Sjögren’s syndrome patients reveals promising tear, saliva and extracellular vesicle disease biomarkers
Source: Arthritis Res Ther. 2019 Jul 31;21:181. doi: 10.1186/s13075-019-1961-4 (PMC6670195; doi:10.1186/s13075-019-1961-4)
Supplement: Supplementary file 8 — Table S5. Upregulated proteins in EVs isolated from whole saliva of non-SS subjects vs. pSS patients. (PDF 206 kb) [file 13075_2019_1961_MOESM8_ESM.pdf]

Table S5. Upregulated proteins in EVs isolated from whole saliva of non-SS subjects vs. pSS patients

Gene name	T-Test (P-Value)	SC non-SS	SC pSS
MUC5A_HUMAN	0,00021	86	312
H31_HUMAN	0,00034	41	69
CD44_HUMAN	0,0016	6	26
CEAM5_HUMAN	0,0016	3	23
TRFL_HUMAN	0,002	572	606
FLNA_HUMAN	0,002	282	312
CIB1_HUMAN	0,0025	3	21
MYH9_HUMAN	0,0029	1353	1282
LG3BP_HUMAN	0,0032	282	263
CFAB_HUMAN	0,0033	12	32
TCPA_HUMAN	0,004	0	9
SPTN1_HUMAN	0,0043	20	54
MVP_HUMAN	0,006	76	107
CD59_HUMAN	0,0063	79	79
LYN_HUMAN	0,0076	56	99
OLFM4_HUMAN	0,0077	180	223
H2B1H_HUMAN	0,0083	164	175
NGAL_HUMAN	0,0084	55	73
FCG3B_HUMAN	0,0088	48	60
NIBAN_HUMAN	0,0093	3	15
1A03_HUMAN	0,0095	0	29
H4_HUMAN	0,01	164	177
LA_HUMAN	0,01	0	6
S10A9_HUMAN	0,012	439	417
G6PD_HUMAN	0,012	109	143
GSHR_HUMAN	0,013	6	18
S10A8_HUMAN	0,014	274	330
HNRPK_HUMAN	0,014	1	8
SIGL9_HUMAN	0,015	0	10
TM11B_HUMAN	0,016	27	51
SAA1_HUMAN	0,016	30	43
PSME1_HUMAN	0,016	0	11
ELNE_HUMAN	0,017	180	207
CYTB_HUMAN	0,017	73	72
KPCB_HUMAN	0,018	4	28
ARP3_HUMAN	0,019	87	101
MUC4_HUMAN	0,019	56	80
RGS19_HUMAN	0,019	23	29
ARP2_HUMAN	0,02	44	66
PADI4_HUMAN	0,021	30	74
IGHG1_HUMAN	0,022	301	303
ECM1_HUMAN	0,022	107	103
TERA_HUMAN	0,022	14	36
IL36A_HUMAN	0,022	15	26
TCPZ_HUMAN	0,023	2	13
MYL6_HUMAN	0,025	145	133

PLS1_HUMAN	0,025	33	46
EF1G_HUMAN	0,025	17	31
GDIR1_HUMAN	0,026	11	21
RO52_HUMAN	0,026	0	8
FCG3A_HUMAN	0,026	0	19
KLK14_HUMAN	0,027	0	7
GIT2_HUMAN	0,027	0	7
PLAC8_HUMAN	0,027	0	7
DYHC1_HUMAN	0,028	12	25
GANAB_HUMAN	0,029	45	75
ATPB_HUMAN	0,029	16	38
CDD_HUMAN	0,029	20	35
CAZA1_HUMAN	0,03	26	36
PDIA6_HUMAN	0,03	0	9
HNRH1_HUMAN	0,03	0	5
CLH1_HUMAN	0,031	141	164
VPS28_HUMAN	0,031	3	11
RL30_HUMAN	0,031	0	5
GYS1_HUMAN	0,032	7	18
DOCK2_HUMAN	0,033	22	44
TSP1_HUMAN	0,033	14	45
PRB2_HUMAN	0,033	8	14
CATG_HUMAN	0,034	550	466
FLOT1_HUMAN	0,034	109	116
BST1_HUMAN	0,035	72	79
ANXA4_HUMAN	0,036	91	95
TOM1_HUMAN	0,036	2	15
QCR1_HUMAN	0,037	0	10
TCPD_HUMAN	0,038	1	12
RALB_HUMAN	0,038	20	30
K1H1_HUMAN	0,039	5	86
ML12A_HUMAN	0,042	61	53
PDIA3_HUMAN	0,042	29	42
SQRD_HUMAN	0,042	15	29
TCPG_HUMAN	0,042	4	15
KT33B_HUMAN	0,043	0	51
VIME_HUMAN	0,044	30	38
MUC2_HUMAN	0,044	0	51
1B07_HUMAN	0,044	0	30
SPR2A_HUMAN	0,045	7	22
RAN_HUMAN	0,046	10	20
SPTB2_HUMAN	0,048	13	24
CAPG_HUMAN	0,049	3	18
