# Supplementary material for: Proteomic and histopathological characterisation of sicca subjects and primary Sjögren’s syndrome patients reveals promising tear, saliva and extracellular vesicle disease biomarkers
Source: Arthritis Res Ther. 2019 Jul 31;21:181. doi: 10.1186/s13075-019-1961-4 (PMC6670195; doi:10.1186/s13075-019-1961-4)
Supplement: Supplementary file 9 — Table S6. Upregulated proteins in EVs isolated from whole saliva of controls vs. pSS patients. (PDF 211 kb) [file 13075_2019_1961_MOESM9_ESM.pdf]

Table S6. Upregulated proteins in EVs isolated from whole saliva of controls vs. pSS patients

Gene name	T-Test (P-Value)	SC controls	SC pSS
MUC5A_HUMAN	0,00038	0	312
OLFM4_HUMAN	0,0012	78	223
FCN1_HUMAN	0,0012	14	46
MUC4_HUMAN	0,0016	18	80
FLNA_HUMAN	0,002	161	312
RGS19_HUMAN	0,002	10	29
CNFN_HUMAN	0,0025	0	18
GNA13_HUMAN	0,0027	8	37
CEAM5_HUMAN	0,0028	0	23
CFAB_HUMAN	0,0039	5	32
CD44_HUMAN	0,005	3	26
FCG3B_HUMAN	0,0054	26	60
SHPS1_HUMAN	0,0058	4	24
TM11B_HUMAN	0,0065	13	51
TRFL_HUMAN	0,0071	374	606
PTPRJ_HUMAN	0,0072	21	64
EZRI_HUMAN	0,0091	120	200
VPS28_HUMAN	0,0094	0	11
LG3BP_HUMAN	0,0095	180	263
CD59_HUMAN	0,0098	50	79
LYN_HUMAN	0,01	26	99
CY24B_HUMAN	0,011	79	139
ARP2_HUMAN	0,011	20	66
GSHR_HUMAN	0,011	1	18
FLOT1_HUMAN	0,012	51	116
GANAB_HUMAN	0,012	20	75
ANXA4_HUMAN	0,013	47	95
ML12A_HUMAN	0,013	34	53
DMBT1_HUMAN	0,015	1098	1856
ARAP1_HUMAN	0,015	0	11
GNAI3_HUMAN	0,016	57	140
HNRPD_HUMAN	0,016	0	13
SAA1_HUMAN	0,017	19	43
ARPC4_HUMAN	0,019	16	46
ANXA1_HUMAN	0,02	405	557
DOCK2_HUMAN	0,02	10	44
DYHC1_HUMAN	0,021	6	25
GBB4_HUMAN	0,021	30	88
IGHG1_HUMAN	0,022	185	303
CIB1_HUMAN	0,022	4	21
SNP23_HUMAN	0,022	0	15
VAT1_HUMAN	0,023	39	67
FIBG_HUMAN	0,024	139	218
CLH1_HUMAN	0,024	79	164
CDC42_HUMAN	0,024	24	66
GDIR1_HUMAN	0,024	6	21

MOES_HUMAN	0,025	120	217
ITIH1_HUMAN	0,025	2	17
LOX5_HUMAN	0,025	0	12
PDCD6_HUMAN	0,025	0	8
G6PD_HUMAN	0,026	67	143
PDIA3_HUMAN	0,027	14	42
COR1A_HUMAN	0,028	96	160
IGHG3_HUMAN	0,028	64	155
MYO1F_HUMAN	0,028	13	46
STEA4_HUMAN	0,028	4	21
CO4A_HUMAN	0,028	15	87
CRNN_HUMAN	0,029	37	71
DNJC3_HUMAN	0,029	1	17
MIF_HUMAN	0,029	0	9
NUCB1_HUMAN	0,029	0	10
HS71A_HUMAN (+1)	0,03	80	151
VA0D1_HUMAN	0,03	2	13
PKHO2_HUMAN	0,031	0	12
GNAI2_HUMAN	0,032	164	287
CAZA1_HUMAN	0,032	17	36
PCBP1_HUMAN	0,032	19	33
CO3_HUMAN	0,033	131	247
ARP3_HUMAN	0,033	57	101
DBNL_HUMAN	0,035	1	8
1A03_HUMAN	0,035	0	29
MYH9_HUMAN	0,036	916	1282
SYTL1_HUMAN	0,037	0	6
TS101_HUMAN	0,037	0	6
LA_HUMAN	0,037	0	6
PDC6I_HUMAN	0,038	76	137
PTN6_HUMAN	0,038	5	29
RABP2_HUMAN	0,038	3	23
ITAX_HUMAN	0,038	2	26
RAP1A_HUMAN	0,038	23	65
APMAP_HUMAN	0,039	20	47
SIGL5_HUMAN	0,039	10	29
TKT_HUMAN	0,04	33	87
TCPA_HUMAN	0,04	1	9
PURA2_HUMAN	0,04	0	9
ARF6_HUMAN	0,04	0	9
PTPRC_HUMAN	0,042	90	183
NCF4_HUMAN	0,042	16	41
RALB_HUMAN	0,042	12	30
PADI4_HUMAN	0,044	17	74
ACSL1_HUMAN	0,044	0	12
S10AG_HUMAN	0,045	8	23
KPCB_HUMAN	0,045	2	28
PRDX5_HUMAN	0,046	10	29
STXB2_HUMAN	0,047	40	81
SLK_HUMAN	0,047	2	14

CAPZB_HUMAN	0,049	47	73
NGAL_HUMAN	0,049	41	73
NHRF1_HUMAN	0,049	30	48
ITIH2_HUMAN	0,049	6	21
